# Multilayer Nanocarrier for the Codelivery of Interferons: A Promising Strategy for Biocompatible and Long-Acting Antiviral Treatment

**DOI:** 10.3390/pharmaceutics16111349

**Published:** 2024-10-22

**Authors:** Thelvia I. Ramos, Carlos A. Villacis-Aguirre, Felipe Sandoval Sandoval, Sarah Martin-Solano, Viana Manrique-Suárez, Hortensia Rodríguez, Leandro Santiago-Padilla, Alexis Debut, Carolina Gómez-Gaete, Marbel Torres Arias, Raquel Montesino, Emilio Lamazares, Ignacio Cabezas, Florence Hugues, Natalie C. Parra, Claudia Altamirano, Oliberto Sánchez Ramos, Nelson Santiago-Vispo, Jorge R. Toledo

**Affiliations:** 1 Biotechnology and Biopharmaceutical Laboratory, Departamento de Fisiopatología, Facultad de Ciencias Biológicas, Universidad de Concepción, Víctor Lamas 1290, P.O. Box 160-C, Concepción 4030000, Chile; carlovillacis@udec.cl (C.A.V.-A.); felisandoval@udec.cl (F.S.S.); vmanrique@udec.cl (V.M.-S.); rmontesino@udec.cl (R.M.); elamazares@udec.cl (E.L.); natparra@udec.cl (N.C.P.); 2 Grupo de Investigación en Sanidad Animal y Humana (GISAH), Departamento de Ciencias de la Vida y la Agricultura, Universidad de las Fuerzas Armadas ESPE, Sangolquí 171103, Ecuador; ssmartin@espe.edu.ec (S.M.-S.); mmtorres@espe.edu.ec (M.T.A.); 3 Yachay Tech Medicinal Chemistry Research Group (MedChem-YT), School of Chemical Science and Engineering, Yachay University for Experimental Technology and Research (Yachay Tech), Yachay City of Knowledge, Urcuqui 100119, Ecuador; hmrodriguez@yachaytech.edu.ec; 4 Max Delbrück Center for Molecular Medicine in the Helmholtz Association, 13125 Berlin, Germany; leandro.santiago@mdc-berlin.de; 5 Laboratory of Characterization of Nanomaterials, Center of Nanoscience and Nanotecnology, Universidad de las Fuerzas Armadas ESPE, Sangolquí 171103, Ecuador; apdebut@espe.edu.ec; 6 Department of Pharmacy, Faculty of Pharmacy, Universidad de Concepción, Concepción 4030000, Chile; cargomez@udec.cl; 7 Clinical Sciences Department, Faculty of Veterinary Sciences, Universidad de Concepción, Vicente Méndez 595, Chillán 3780000, Chile; oscabeza@udec.cl (I.C.); flohugues@udec.cl (F.H.); 8 Escuela de Ingeniería Bioquímica, Facultad de Ingeniería, Pontificia Universidad Católica de Valparaíso, Av. Brasil 2085, Valparaíso 2362803, Chile; claudia.altamirano@pucv.cl; 9 Laboratory of Recombinant Biopharmaceuticals, Departamento de Farmacología, Facultad de Ciencias Biológicas, Universidad de Concepción, Víctor Lamas 1290, P.O. Box 160-C, Concepción 4030000, Chile; osanchez@udec.cl; 10 Bionatura Journal, Clinical Biotec SL, 28029 Madrid, Spain; santiago@clinicalbiotec.com

**Keywords:** nanoencapsulation, interferons, antiviral, antiproliferative, immunoregulation, drug delivery system, core-shell, toxicity

## Abstract

**Background:** Interferons (IFNs) are cytokines involved in the immune response with a synergistic regulatory effect on the immune response. They are therapeutics for various viral and proliferative conditions, with proven safety and efficacy. Their clinical application is challenging due to the molecules’ size, degradation, and pharmacokinetics. We are working on new drug delivery systems that provide adequate therapeutic concentrations for these cytokines and prolong their half-life in the circulation, such as nanoformulations. **Methods:** Through nanoencapsulation using electrospray technology and biocompatible and biodegradable polymers, we are developing a controlled release system based on nanoparticles for viral infections of the respiratory tract. **Results:** We developed a controlled release system for viral respiratory tract infections. A prototype nanoparticle with a core was created, which hydrolyzed the polyvinylpyrrolidone (PVP) shell , releasing the active ingredients interferon-alpha (IFN-α) and interferon-gamma (IFN-γ). The chitosan (QS) core degraded slowly, with a controlled release of IFN-α. The primary and rapid effect of the interferon combination ensured an antiviral and immunoregulatory response from day one, induced by IFN-α and enhanced by IFN-γ. The multilayer design demonstrated an optimal toxicity profile. **Conclusions:** This formulation is an inhaled dry powder intended for the non-invasive intranasal route. The product does not require a cold chain and has the potential for self-administration in the face of emerging viral infections. This novel drug has applications in multiple infectious, oncological, and autoimmune conditions, and further development is proposed for its therapeutic potential. This prototype would ensure greater bioavailability, controlled release, fewer adverse effects, and robust biological action through the simultaneous action of both molecules.

## 1. Introduction

Emerging or re-emerging diseases are defined as newly identified and unknown viral infections or the re-emergence of a known communicable disease following a significant decline in incidence [1,2]. Seventy percent of emerging infections in humans are zoonotic in origin [2] and pose a challenge to health systems [3], and the antiviral therapies used are insufficient [4]. Emerging or re-emerging viruses that have mutated may be resistant to the effects of direct-acting antivirals, while vaccines require the identification of the viral strain before the production stage, precluding their use at the onset of any new outbreak [5]. Against this background, there is a need to develop preventive and therapeutic antiviral therapies that allow the activation of the first lines of defense, particularly the innate immune response, that can be used generally in emerging and re-emerging infections [6]. The first organized defense that a viral pathogen must overcome during infection is the host’s innate immune response, which involves several effector mechanisms such as type I interferons, the complement cascade, NK cells, apoptosis, autophagy, and the Toll-like receptor (TLRs) pathway [7]; the adaptive immune system is activated and primed to respond appropriately [8].

Interferons are a family of cytokines whose functions have been known for over six decades [9]. The main activity associated with these proteins is stimulating the immune system, triggering antiviral, antiproliferative, and immunomodulatory responses [10]. The antiviral mechanism of IFNs is based on the control of gene expression [11]. They are cell signaling glycoproteins, effectors of innate and adaptive immunity, associated with activating humoral and cellular responses against different pathogens derived from a neoplastic process or other damage responses to the organism [12].

The synergistic and combined response of both interferons has been demonstrated in several studies aimed at controlling respiratory viral infections [13,14,15]. Multiple waves of type I IFN production, starting with IFN-β at 12 h post-infection, are responsible for the induction of inflammatory monocyte recruitment, leading to IFN-γ production in NK cells induced by interleukin 18 (IL-18) [16]. At 48 h post-infection, there is increased production of type I IFNs (IFN-α and IFN-β) and an increase in IFN-γ released by NK cells, which promotes APC maturation, positive regulation of co-stimulatory molecules, processing, and antigen presentation toward Th1 polarization [17], while simultaneously suppressing innate lymphoid cell-mediated immunopathology (ILC2) [18].

Despite the recognized efficacy of biological activity, these cytokines have limited activity as therapeutics due to the size of the molecules, sensitivity to degradation, elimination by the reticuloendothelial system (RES), rapid renal clearance, and toxicity at high doses [19,20].

Currently, research for these biotherapeutics focuses on obtaining new delivery systems that provide adequate therapeutic concentrations, lower toxicity, and better active ingredient protection [21,22]. Researchers worldwide have evaluated the possibility of encapsulating IFNs such as PEGylation, liposomes, micellar systems, self-assembled nanostructures, microparticles, and nanoparticles, be they metallic, polymeric, or hybrid [23,24]. The most successful form of encapsulation for these proteins reported in the literature is nanoparticle systems (NPs), as nanoformulations can improve the therapeutic index, especially in IFNs with a short half-life, which require frequent administration at high doses [25,26]. Nanoparticles are nanoscale structures that can be either capsules or spheres, depending on their internal constitution [27]. These systems simplify the administration of IFNs, improve therapeutic efficacy, and reduce dose-related side effects without decreasing biological activity or altering the protein structure [28]. The development of new delivery strategies allowing local and controlled release of IFN-α through biological activity potentiated with another cytokine that regulates its action, as is the case with IFN-γ, would allow achieving both an optimal preventive and therapeutic response to immune system activation [29]. This innovative approach uses biocompatible and biodegradable polymers to achieve a controlled release, a prolonged half-life, selective delivery, and an optimal toxicity profile using nanoencapsulation [30].

We proposed an antiviral prototype to prevent or treat respiratory tract viral infections. After application, the formulation will be administered nasally, generating a prolonged protective effect. Entering numerous viral respiratory tract infections will activate the mucosal immune response. The nasal mucosa provides a first-line defense against inhaled pathogens as an epithelial barrier to most infectious agents, especially respiratory viruses [31]. Intranasal administration offers several advantages, such as low invasiveness, contact with highly vascularized absorption surfaces, low proteolysis, retention time on the mucosal surface, increased epithelium penetrability, and reduced drug metabolic degradation [32]. The selection of a nanoencapsulation system for drug delivery improves solubility, stability, and controlled release, and its gradually improved functions suppress the shortcomings of other delivery systems [33,34]. For example, in the nasal cavity, there are cells in the upper respiratory tract, microplastic cells, which transport antigens across the mucosa, and nanoparticles that, due to their small size, manage to enter microfold cells favoring the penetration and bioavailability of the drug [33,35].

For this reason, nanoparticles overcome the physical barrier of the mucosa and maintain a prolonged retention time on the cell surface, penetrating effectively and accumulating on the epithelial surface; in addition, they protect the active ingredient from biological and chemical degradation [36]. It has been suggested that nanoparticles can be combined with absorption enhancers and functional excipients to improve penetrability at biological barriers by temporarily opening intercellular tight junctions [37]. In addition, a delivery system that does not induce side effects is the first element to be considered in the search for a formulation. Among the polymeric materials that meet this requirement are polyvinylpyrrolidone [38] and chitosan [39]. Polyvinylpyrrolidone is a biocompatible polymer that does not produce cytotoxicity [38]. The presence of hydroxyl and carbonyl groups in the structure allows better retention of PVP in the nanoparticles; the polymer chains of this compound separate very quickly in the presence of water, releasing the encapsulated products [40]. There are several applications of PVP with proteins and especially with interferons for mucosal administration (genital and nasal) because it is a biologically compatible polymer, a facilitator of absorption, and an antioxidant [41,42,43]. Chitosan is a natural polysaccharide that has been used in the development of core-shell structures [44]. It is a biodegradable non-toxic polycation with biological properties such as biocompatibility, antimicrobial activity, healing, low immunogenicity, and low skin irritation [45]. Since 2001, QS has been approved by the US Food and Drug Administration as a GRAS (generally recognized as safe) substance [46], and its biological properties justify applications in biomedicine [47].

We developed a nanoformulation with antiviral activity and core-shell structure using recombinant alpha and gamma interferons, produced at a laboratory scale with high purity. Biocompatible and biodegradable polymers were used as encapsulating matrices in a combination that had not been previously developed. Once the particles come into contact with the nasal mucosa, they undergo hydrolysis and degradation. This will result in the release of the products in the external layer, followed by the degradation of the internal polymeric layer. The technique for synthesizing the nanoparticles was coaxial electrospraying, which allows the production of multilayer particles ranging from 10 to 100 µm by applying a high electric field between the coaxial capillary injector and the collector [48]. This prototype aims to respond rapidly to emerging and re-emerging respiratory viral infections for which no effective therapeutic options exist. The scientific and infrastructural basis for formulating these molecules is available to reduce the high morbidity and mortality rates of respiratory viral infections. The antiviral prototype has the potential to facilitate emerging therapies.

## 2. Materials and Methods

### 2.1. Materials

#### 2.1.1. Interferons as Active Ingredients

Recombinant interferon-alpha 2b (rhIFNα-2b) and recombinant interferon-gamma (rhIFN-γ), used as active principles of the formulation, were produced in the *E. coli* SHuffle^®^ T7 Express strain and transformed with the expression vectors pET22b-rhIFNα-2b and pET22b-rhIFN-γ, respectively. The identity of both plasmids was verified by automated sequencing performed by Macrogen Company (Seoul, South Korea).

rhIFNα-2b Sigma-Aldrich (St. Louis, MO, USA) [49] and rhIFN-γ Thermo Fisher Scientific (Waltham, MA, USA) [50] were used as standards.

#### 2.1.2. Encapsulation Materials

Low molecular weight (LMW) QS (50,000–190,000 Da), deacetylated chitin, Poly D-glucosamine, LMW PVP (40,000 Da), and absolute ethanol (EMPLURA^®^) were purchased from Sigma-Aldrich (St. Louis, MO, USA).

#### 2.1.3. Cell Lines

HEp-2 (human laryngeal carcinoma, ATCC CCL-23), HeLa (derived from cervical carcinoma of human origin, ATCC CCL-2), and HFF (human foreskin fibroblasts, ATCC SCRC-1041) cell lines were obtained from ATCC (American Type Culture Collection, Manassas, VA, USA).

HFF and HEp-2 cell lines were cultured in Dulbecco’s Modified Eagle Medium (DMEM) supplemented with 15% *w*/*v* fetal bovine serum (FBS) and penicillin 100 IU/mL and streptomycin 100 μg/mL. HeLa cells were cultured in Eagle’s Minimum Essential Medium (EMEM) supplemented with 10% *w*/*v* SFB, 100 IU/mL penicillin, and 100 μg/mL streptomycin. Cells were incubated at 37 °C in a humidified atmosphere with 5% CO_2_.

#### 2.1.4. Animal Models

Adult rabbits (*Oryctolagus cuniculus)* were of the same sex with a body mass of more than 2.5 kg.

Sheep (*Ovis aries*) were aged 3 to 6 months, had a body weight 39 to 64 kg, and were sex-indistinct.

The Comité de Ética, Bioética y Bioseguridad of Universidad de Concepción approved all animal studies (CEBB 1370-2023). All experiments were conducted in compliance with institutional and national guidelines.

### 2.2. Synthesis of Core-Shell NPs with Interferons Alpha and Gamma

Nanoparticles with an empty core/shell structure (CS NPs) and those encapsulating rhIFNα-2b and rhIFN-γ were prepared by electrospraying. We prepared two polymer solutions: one for the outer shell of the formulation and one for the core. We employed 10% (*w*/*v*) PVP dissolved in ethanol, and rhIFNα-2b and rhIFN-γ (1 mg/mL each) as active principles for the outer shell [51]. In the core, we used an active principle of QS 1% (*w*/*v*) dissolved in 0.5% acetic acid and rhIFNα-2b (2 mg/mL) [52]. Two 10 mL plastic syringes (NIPRO Europe, Mechelen, Belgium) were used to load each solution in independent pumps in the electrospraying Professional Lab Device (DOXA Microfluidics, Málaga, Spain). Flow rate (0.5–2 mL/h) and voltage difference (15–30 kV) values were set at a temperature of 16 °C and a relative humidity of 40%. The Taylor cone formation was observed with IC Capture v2.4 software (The Imaging Source, Charlotte, NC, USA). We collected the nanoparticle powder with the help of a brush from the flat collector coated with metallic paper and stored it in a sterile microtube previously weighed [53].

Chitosan NPs (QS NPs), which constitute the core of the CS formulation, were synthesized in two forms: QS empty and QS encapsulating rhIFNα-2b, as described for CS NPs. These unshelled particles were employed as controls for the efficiency of the CS NPs encapsulating rhIFNα-2b and rhIFN-γ in the in vitro physicochemical and biological characterization experiments (see Supplementary Information).

### 2.3. Physicochemical Characterization of Nanoparticles

#### 2.3.1. Morphology and Size

Morphological characterization and size estimation of the NPs were performed using a Field Emission Scanning Electron Microscope (FE-SEM, TESCAN MIRA 3, Brno, Kohoutovice, Czech Republic), resolution 1.2 nm and 30 kV. Micrographs were obtained with a secondary electron (SE) detector, Scanning Transmission Electron Microscopy (STEM), and Scanning Electron Microscopy (SEM) in transmission mode, brightfield (TE-BF), and darkfield (TE-DF). For SEM visualization, the nanoparticles were placed on a double-sided carbon tape and coated with gold for 60 s. In STEM visualization (FE-SEM), ethyl acetate was selected as the dispersant for PVP. Approximately 5 to 10 μL of each suspension was placed on a copper grid (300 grid square, 63 µm side square) for Formvar/carbon Transmission Electron Microscopy and proceeded with FE-SEM observation.

Histograms of the diameters were created from the SEM and STEM micrographs from each sample with the open source software FIJI© v1.53f51 and with the software SCILAB© v6.0.2 (Dassault Systèmes, Vélizy-Villacoublay, France). The probability density function (PDF) generated by Matlab© vR2022a software (Mathworks Inc., Natick, MA, USA) was also determined. Using the fitdist function, measurements ranging from 450 nm [54] were selected, and the values were fitted to a normal distribution.

Particle size estimation was also performed by dynamic light scattering (DLS) using the Malvern Zetasizer Nano ZS90 (Malvern Panalytical, Malvern, UK), equipped with a He-Ne 633 nm laser that determined the polydispersity index and zeta potential. The nanoparticles were dispersed in water and transferred to Malvern DTS0012 disposable polystyrene cuvettes for size estimation and to DTS1070 cuvettes to determine the zeta potential of the particles [55].

#### 2.3.2. Encapsulation Efficiency

This parameter was determined by molecular exclusion high-performance liquid chromatography (SEC-HPLC) using the ÄKTA Start chromatography system (GE Healthcare Life Sciences, Uppsala, Sweden). The amount of free rhIFNα-2b and rhIFN-γ in the supernatant of the nanoparticles was quantified using TSK gel matrix G2000SW (7.5 mm ID × 60 cm × 2; Tosoh Bioscience GmbH, Griesheim, Germany) [47]. Recombinantly produced rhIFNα (8 mL, 30 μg/mL in PBS) and rhIFN-γ (8 mL, 27 µg/mL in PBS) were used as the positive control, and empty NPs were used as the negative control to measure the total amount of protein and polymer interference, respectively. Furthermore, an additional control was the QS NPs formulation, which constituted the core of the CS NPs. Samples of the CS NPs and their control, the QS NPs, were dissolved independently in 500 µL of 1% acetic acid by gentle shaking for 30 min and one h (150 rpm) (WiseShaker orbital shaker, PMI Labortechnik, Wettingen, Germany) at 37 °C. Samples were centrifuged at 9600× *g* (Thermo Scientific Legend Micro 21 Centrifuge, Thermo Fisher Scientific, Waltham, MA, USA) for 20 min, and the supernatant was collected for the column run. In total, 20 µL of each sample was passed through the matrix at a 1 mL/min flow rate in a solution of PBS buffer and 300 mM NaCl (mobile phase). From the chromatogram obtained from the absorbance at 280 nm, the areas under the curve of each formulation element were quantified. The encapsulation efficiency (EE) was calculated with Equation (1), as follows:

(1) $$\%EE=\frac{{IFN}_{total}-{IFN}_{non-encapsulated}}{{IFN}_{total}}*100$$

#### 2.3.3. Fourier Transform Infrared Spectroscopy

Fourier Transform Attenuated Total Reflectance Fourier Transform Infrared Spectroscopy (ATR-FTIR) was used [47,56] to study and compare the chemical composition of the NPs, identifying characteristic functional groups in vacuum lyophilized nanoparticle samples loaded with the interferon molecules (rhIFNα-2b and rhIFN-γ). The nanoparticles were centrifuged at 9600× *g* for 30 min, and the pellets were dispersed in PBS and freeze-dried under a vacuum to remove water molecules. All FTIR spectra were performed for the dried samples in the 400–4000 cm^−1^ region using Agilent Cary 630 FTIR-ATR spectrophotometer (Agilent Technologies Inc., Santa Clara, CA, USA), and the data were analyzed using OMNIC vINQSOF018 software (Thermo Fisher Scientific, Waltham, MA, USA).

#### 2.3.4. Differential Scanning Calorimetry Analysis

Samples corresponding to PVP (40,000 Da) and QS LMW (50,000–190,000 Da) polymers, plus CS NPs encapsulating rhIFNα-2b/rhIFN-γ, empty CS NPs, and controls corresponding to the core of the CS formulation, were analyzed using Differential Scanning Calorimetry (DSC). The different thermal transitions presented in the samples were detected using a Simultaneous Thermal Analyzer (STA) 8000 (PerkinElmer Inc., Waltham, MA, USA). Overall, 5–10 mg of the samples were placed in hermetically sealed empty aluminum trays. The sample was heated from 25 °C to a temperature of 270 °C with a heating rate of 20 °C/min [57,58].

#### 2.3.5. In Vitro Kinetic Release Study

The release profile of the proteins was determined using an experiment simulating human physiological conditions [59]. The assay was conducted using CS NPs, rhIFNα-2b and rhIFN-γ, and the control, representing the CS formulation’s core. In total, 200 mg of each formulation was resuspended in 5 mL of physiological saline at pH 6.8 (pH of the nasal mucosa). The amount of protein included according to the NPs in the respective polymer solution (core 2 mg/mL of rhIFNα-2b and shell 1 mg/mL of each protein), the EE result, and the concentration of each protein were also considered. Once the NPs were reconstituted with saline at pH 6.8, the tubes were incubated using slow shaking at 150 rpm (WiseShake Wisd Laboratory Instrument orbital shaker, Avantor, Austria) at 37 °C. In total, 300 µL per day of the NPs were subtracted and centrifuged at 9600× *g* for 30 min, and the supernatant was separated from the pellet until 14 days were completed. The supernatant was recovered from each tube and stored at −20 °C until the end of the assay. The protein concentration of each day was quantified using the Micro BCA Protein Assay Kit (Thermo Fisher Scientific, Waltham, MA, USA), according to the manufacturer’s protocol [60]. The absorbance at 570 nm was measured on the Synergy HTX multimode microplate reader (Agilent Technologies Inc., Santa Clara, CA, USA).

### 2.4. Biological Characterization of Nanoparticles

#### 2.4.1. Effect of Encapsulated Active Ingredients on Cell Viability

Cell viability was determined in two cell lines, HeLa and HFF, incubated at different concentrations of the NPs, using the MTT (3-(4,5-dimethylthiazol-2-yl)-2,5-diphenyl tetrazolium bromide) assay [61]. The HeLa cell line was used because of the activity of IFN-α in inhibiting its proliferation (apoptosis and antiproliferative activity of encapsulated interferons) [62]. Cells were seeded in 96-well culture plates (Corning™ Costar™, Thermo Fisher Scientific, Waltham, MA, USA) at a concentration of 10^4^ cells/well and incubated in DMEM medium with 10% SFB at 37 °C 5% CO_2_ for 24 h. Several dilutions of the nanoformulation were prepared from a 4 mg powder dispersion in 100 µL of DMEM medium with 10% SFB. Overall, 100 µL per well was added to the plates seeded with the cells. These were incubated at 37 °C and 5% CO_2_ for 24 h. Wells with untreated cells were used as a 100% cell viability control. Cells with 400 µM CoCl_2_ and 30% H_2_O_2_ were used as a cytotoxicity control. After incubation, the cells were examined under an inverted microscope to visualize the cell density of the lines at the different dilutions, and micrographs were taken (MCX1600, MICROS Company, Sundew, Austria). The culture medium was then removed and washed with PBS, and 100 µL of fresh medium and 10 µL of MTT solution (5 mg/mL) were added. The plate was incubated in the dark (37 °C, 5% CO_2_, three hours), and over this solution, 100 µL dimethyl sulfoxide (DMSO) was added to dissolve the crystals for 15 min at room temperature. Finally, the Absorbance of the plate was read at 570 nm in a Multiskan GO spectrophotometer (Thermo Fisher Scientific, Waltham, MA). The data were analyzed and plotted using Prism v8.0 software (GraphPad Software, Boston, MA, USA). The normalized absorbance data were checked with the Shapiro–Wilk test to ensure they conform to a normal distribution. We assessed cell viability using ANOVA and multiple comparison tests to determine which concentrations significantly modified cell viability.

#### 2.4.2. In Vitro Antiviral Biological Activity

The antiviral activity of the encapsulated proteins with their respective commercial reference standards was measured in HEp-2 cells by inhibition of the cytopathic effect of the Mengo virus to establish the maximum effective mean concentration (EC_50_) [63,64,65].

Cells were seeded in 96-well plates (Corning™ Costar™, Thermo Fisher Scientific, Waltham, MA, USA) at 1.5 × 10^4^ cells/well in DMEM + SFB 5% + neomycin (100 IU/mL) and incubated at 37 °C and 5% CO_2_ for 24 h. Cells were treated with CS NPs encapsulating rhIFNα-2b and rhIFN-γ, empty CS NPs, and controls corresponding to the core CS formulation (empty QS NPs and QS NPs +rhIFNα-2b). In total, 1 mg of each formulation was weighed and dispersed in 1 mL of DMEM SFB 2% + neomycin (100 IU/mL). Dilutions of each formulation were made to a stock of 100 ng/mL and, as a control, 100 μL of DMEM + SFB 2% medium. The medium was replaced with 100 µL of Mengo virus in DMEM + SFB 2%, and the plates were incubated for 24 h at 37 °C and 5% CO_2_. The plates were washed, fixed, and stained with 0.5% and 20% crystal violet solution. The crystal violet solution was dissolved with 10% acetic acid. The plate absorbance reading was performed at 590 nm on a Synergy HTX Multimode Reader spectrophotometer (Agilent Technologies Inc., Santa Clara, CA, USA).

To obtain a sigmoidal curve and calculate the EC_50_, initial concentrations of the rhIFNα-2b standard were worked from 0.33 μg/mL and for the rhIFN-γ standard 0.033 μg/mL from a solution at 500 µg/mL, performing serial dilutions 1:5. Cell control (cc) wells with cells without Mengo virus were estimated as cell control (cc), and cells without interferon treatment exposed to the infectious agent were estimated as virus control (cv). Using the Equation (2), the data were fitted to a sigmoid curve to determine the EC_50_ value.

(2) $$Abs\;norm=\frac{Abs-cv}{cc-cv}$$

Considering this, the interferon titer was calculated according to the Equation (3), as follows:

(3) $$IFN\;titer\left(\frac{IU}{mL}\right)=\frac{Sample\;titer}{STD\;titer}*STD\left(\frac{IU}{mL}\right)$$

And the specific activity with Equation (4), as follows:

(4) $$Specific\;activity\left(\frac{IU}{mL}\right)=\frac{IFN\;titer\;(IU/mL)}{IFN\;concentration\;(mg/mL)}$$

The data were fitted to a sigmoid curve that determined the value of the EC_50_, the dilution that generated 50% cell death, and the titer and specific activity of the encapsulated interferons.

#### 2.4.3. Monolayer Study of the Interaction of NPs by Confocal Microscopy

The behavior of CS NPs encapsulating interferon alpha and gamma interferon in interactions with live cells, specifically HEp-2 cells, was analyzed by confocal microscopy. Only the rhIFNα-2b protein was framed as it was represented in the shell and core of the NPs. At a concentration of 250 μg/mL, HEp-2 cells were treated for 24 h.

The rhIFNα-2b was conjugated to fluorescein 5-isothiocyanate (FITC) [66]. The rhIFNα-2b protein (2 mg/mL) was dissolved in sodium bicarbonate buffer (0.1 M, pH 9) and mixed with FITC (1 mg/mL) in DMSO at a rhIFNα-2b: FITC ratio of 12.5:1 (*v*/*v*). The mixture was placed for 12 h on gentle agitation, protected from light, and unconjugated FITC was removed by dialyzing for 48 h in PBS in a 3.5 kDa cellulose dialysis tube (Thermo Fisher Scientific, Waltham, MA, USA). The rhIFNα-2b-FITC conjugate was used to obtain a batch of tagged protein-loaded NPs [67]. The rhIFNα-2b-FITC conjugate HEp-2 cells were cultured in DMEM medium with 10% (*v*/*v*) SFB 10% (*v*/*v*) penicillin 100 IU/mL and streptomycin 100 μg/mL at 37 °C and 5% CO_2_ (*v*/*v*) and incubated for 24 h. Treatments were plated as follows: empty CS core-shell NPs and CS rhIFNα-2b + FITC core-shell NPs at 250 nm/mL and as a negative control HEp-2 cells in DMEM medium, SFB 1% (*v*/*v*), and 5% CO_2_ (*v*/*v*) and incubated for 24 h.

After a confluence of 50 × 10^4^ empty CS NPs and CS NPs rhIFNα-2b + FITC (green fluorescence) and as a negative control, HEp-2 cells in DMEM medium were applied as treatments. At 24 h after treatment, nuclei were stained with Hoechst 33342 cell nuclei (Invitrogen, USA) dye (blue fluorescence) at a concentration of 5 μg/mL and the mitochondria MitoTracker^®^ Red CMXRos-M7512 marker (Invitrogen, Carlsbad, CA, USA) (red fluorescence) was used at 250 nM.

Cells were incubated for 25 min at 37 °C in dark conditions, washed with PBS, and fixed with 2% paraformaldehyde (PAF) for 5 min, then 15 min at 4% PAF. Cells were plated on slides with mounting medium (VECTASHIELD^®^ antifade DAP, SeraCare KPL, Milford, MA, USA). Upon completion, non-retained dyes were removed using three washing steps with DMEM. Cell fluorescence images were obtained with a Laser Scanning Biological Microscopes Fluoview 2000 Confocal Microscope (Olympus, Melville, NY, USA) [47].

#### 2.4.4. Formulation Stability Under Accelerated Conditions

The stability of the CS NPs + rhIFNα-2b and rhIFN-γ formulations and the control (QS NPs encapsulating rhIFNα-2b), which correspond to the core of the CS formulation, was evaluated under accelerated conditions for 18 days at different temperatures: 4 °C, 16 °C, 25 °C, 30 °C, and 37 °C and in different incubators (ECOCELL 55-ECO, MMM Medcenter Einrichtungen GmbH, München, Germany) [68]. Antiviral activity in HEp-2 cells was also determined by inhibition of the cytopathic effect of the Mengo virus. We weighed 1 mg of each NP, dissolved in 1 mL of DMEM supplemented with 5% SFB + neomycin (100 IU/mL), and serial dilutions up to a stock of 100 ng/mL were performed to plates seeded with HEp-2 cells. We used 100 µL of DMEM + SFB 2% medium as a control. The data were fitted to a sigmoid curve, and the EC_50_ value was determined. Using IFNα-2b and IFN-γ standards as reference, interferon titer, specific activity, and concentration were determined for the formulations.

#### 2.4.5. In Vitro Statistical Analysis

Morphological characterization of the NPs: We determined whether the particle size variability of all formulations had a normal distribution using the Shapiro–Wilk test [69]. A significance level of *p* < 0.05 was used.

Evaluation of the cell viability of the nanoformulations: We used Students’ *t*-test statistics to evaluate the differences between empty and encapsulated formulations, which were considered independent samples. For the overall comparison between the solutions in each group, a one-way repeated measures ANOVA was used. A pairwise comparison was run, considering the Bonferroni correction to correct for type I error. The significance level set was 0.05. All variables were processed with the SPSS 25.0 statistical program and GraphPad Prism v8.0 software.

### 2.5. Evaluation of Initial Toxicity in Vivo

#### 2.5.1. Study of the Mucosal Irritant Potential of the CS Formulation in Rabbits

This study aimed to evaluate the product’s potential to cause irritation to the nasal mucosa and to observe its toxic effects on the macroscopic and microscopic structures within the same tissue [70]. Adult rabbits of the same sex (males) that were clinically healthy, with a body mass between 1.4 and 4 kg, were randomly grouped into 5 experimental groups of 5 animals each. The route of administration selected was intranasal, where inoculations were performed with an equal volume in each group. Group II Placebo (saline) as Group III (empty NPs) constituted controls for Group IV CS NPs (rhIFNα-2b-rhIFN-γ) and Group V (interferons in solution). Total number of animals: 25.

- Group I: control, not treated.
- Group II placebo, saline (NaCl 0.9%, sterile), 50 µL in each nostril (total 100 µL).
- Group III Empty CS NPs, powder formulation, 50 µg dose in each nostril (total 100 µg).
- Group IV NPs CS rhIFNα-2b and rhIFN-γ, powder formulation, 50 µg dose in each nostril (total 100 µg).
- Group V rhIFNα-2b and rhIFN-γ in saline, 50 µL dose in each nostril (total 100 µL).

##### The Procedure of Nanoparticle Administration

The animals were kept supine without sedation so that the product reached the nasal cavity. In a 1 mL syringe, a catheter was attached, loading 50 µg powder of the formulation for each nostril and 50 µL for each nostril in the case of liquid formulations. We performed intranasal administration every twenty-four hours for three consecutive days, and the procedure was repeated for four weeks for 28 applications. We evaluated the condition of the nasal mucosa twenty-four hours after the initial application and every two days after that. In the nasal passages, secretions, dryness, obstruction, respiratory difficulties, erythema, irritation or edema, and behavioral changes were observed until the trial’s end. Body weight and temperature were determined at baseline and during treatment. The rabbits were euthanized (on the last day of the fourth week) to complete the histological analysis. TIVA (total intravenous anesthesia) general anesthesia was administered using a neuroleptanalgesia Xylazine 2% at a dose of 20 mg/kg, associated with Ketamine 10% at a dose of 5 mg/kg. Once the surgical plane was established, a dose of 20 to 40 mg of Mepivacaine 2% was administered percutaneously in the cisterna magna with a response 30 s after the application with cardiorespiratory arrest.

##### Histopathological Study

The nasal mucosa was dissected free, opened longitudinally, and examined for signs of irritation, epithelial tissue damage, or necrosis according to standard necropsy technique for animals [71]. Nasal mucosa samples corresponding to the turbinates were taken completely and fixed in 10% buffered formalin for five days. The samples were retroceded, obtaining a complete cross-section of each nasal turbinate for histological analysis. The samples were dehydrated using a battery of ethanol at different percentages (70, 80, 95, and 100%), rinsed with xylol, and embedded in solid kerosene using a Citadel 1000 tissue processor (Thermo Fisher Scientific, Waltham, MA, USA). Kerosene blocks were assembled using a Microm AP280-1 Inclusor (Wazobia Enterprise, Houston, TX, USA). Finally, the blocks were cut to a thickness of 4 μm using a RM2045 rotation microtome (Leica Biosystems, Nussloch, Germany) and stained with hematoxylin–eosin (Merck laboratory reagents), according to the protocol standardized by the Histopathology Laboratory of the Department of Pathology and Preventive Medicine of the Faculty of Veterinary Sciences of the Universidad de Concepción.

##### Statistical Analysis of Initial In Vivo Rabbit Toxicity Assessment

We used mean and standard deviation to summarize the quantitative variables. The Shapiro–Wilk test determined the normal distribution of weight, temperature, mucosal status, and organ weights. The treatment groups were compared through a one-way ANOVA for the body weight and temperature variables at two-ways baseline and endpoint. A paired analysis of the correlation between the times of the variables body weight and temperature was performed through repeated measures ANOVA. We applied a student’s *t*-test for dependent samples in each treatment group for the two-by-two comparisons. Bonferroni error correction was taken into consideration. The statistical significance set was α = 0.05. For the variable temperatures, the different treatment groups were compared at two-day intervals utilizing a rank test (Sign Test), dependent or paired samples. For organ weights, a relationship was established between the different treatment groups and each of the evaluated organs, applying a one-way ANOVA. The statistical significance set was α = 0.05. All variables were processed using the statistical programs SPSS 25.0, STATISTICA 6.0, and GraphPad Prism v8.0 software.

#### 2.5.2. Study of Release Kinetics and Biological Activity in Sheep

The study aimed to determine the toxicity of the CS core-coated NPs formulation encapsulating the two interferons in a higher organism animal model by establishing mucosal irritability in sheep [72]. Clinically healthy same-sex adult ewes (females) with a body mass between 39 and 66 kg were selected. The ewes were randomly grouped into four experimental groups of 4 animals each, receiving a single dose of treatment applied in the nostrils. The experimental procedures with the animals were adequate so as not to generate suffering or pain, with daily observations and measurements to detect alterations, stress, or animal suffering, which would lead to stopping the trial.

- Group I: control saline solution (NaCl 0.9%, sterile) 2 mL for each nostril.
- Group II Placebo 2 mg of empty powdered CS NPs per nostril.
- Group III NPs CS/ rhIFNα-2b and rhIFN-γ, 2 mg powder per nostril.
- Group IV IFN-α and IFN-γ solution, 2 mL of the solution per nostril.

##### The Procedure of Nanoparticle Administration

Samples were taken from the nasal vestibule by 4-mm punch biopsy on days 4, 8, 12, and 16 in each ewe. Samples were obtained from one nostril, leaving the other nostril for the subsequent evaluation, allowing tissue recovery between one sample collection and the next. Before the biopsy, the animals were locally anesthetized with infiltrative blockade of the facial nerve with 0.5 mL of Mepivacaine as an analgesic and Xylazine 2%, 0.1 mg/kg tranquilizer. The specimen was kept in Eppendorf tubes containing 10% buffered formaldehyde solution (histopathological study).

##### Histopathological Study

The samples were placed in an embedding cassette, and a dehydration process was performed with ethanol of ascending grade (70, 80, 95, and 100%). We rinsed samples with xylol and embedded them in kerosene using a Citadel 1000 tissue processor (Thermo Fisher Scientific, Waltham, MA, USA). Kerosene blocks were assembled using a Microm AP280-1 (Wazobia Enterprise, Houston, TX, USA). The obtained blocks were cut with a Leica rotation microtome model RM2045 (Germany) to a thickness of 4 μm and stained with hematoxylin–eosin (Merck laboratory reagents), according to the standardized protocol of the Histopathology Laboratory of the Department of Pathology and Preventive Medicine of the Faculty of Veterinary Sciences of the Universidad de Concepción. A rhinoscopy was also performed to assess the physiological state of the nasal mucosa with a FUJINON Fiberscope FS-100ER (Fujifilm, Japan) before the biopsies, at different times: at the beginning, at 14 days, and at the end of the treatment. We used GraphPad Prism v8.0 software to obtain the graphs and perform the statistical analysis.

##### Statistical Analysis of Initial In Vivo Sheep Toxicity Assessment

The mean and standard deviation variables were summarized in the statistical analysis of the study of release kinetics and biological activity in sheep. The normal distribution was determined for weight, temperature, and nasal mucosa status using the Shapiro–Wilk test. Treatment groups were compared through a one-way ANOVA for the variables body weight and temperature at baseline between the different treatment groups and at the end. We performed a correlation analysis through a paired analysis between times for the variables body weight and temperature employing repeated measures ANOVA and for the two-by-two comparison in each treatment group through a student’s *t*-test for dependent samples. A Bonferroni error correction was considered as there were more than two evaluations. The statistical significance set was α = 0.05. For the temperature variable, a Sign Test for dependent or paired samples was also performed to compare the temperatures of the different groups of treatments evaluated at two-day intervals. All variables were processed using the statistical programs SPSS 25.0, STATISTICA 6.0, and GraphPad Prism v8.0 software.

## 3. Results and Discussion

Polymeric nanoparticles were created by combining IFN-α and IFN-γ with a hydrolyzable polyvinylpyrrolidone shell, and a low molecular weight chitosan encapsulates the IFN-α as the active principle in a core-shell design. Adjusting the polymeric formulations’ pH (away from the isoelectric point) and the proteins were required to obtain the nanoparticles. It has been described that interferon alpha is labile in acidic media and shows structural changes unfolding below pH 4; the protein exhibits maximum conformational stability at pH 7 [73]. This correction facilitated the formation of the NPs in the solid state, evaporating the solvent during the synthesis process, becoming an advantage over other encapsulation methods (such as double emulsion) that require additional solvent removal steps [74].

Empty CS NPs and CS NPs encapsulating rhIFNα-2b and rhIFN-γ, consisting of 10% (*w*/*v*) BPM PVP (40,000 Da) for the shell and 1% (*w*/*v*) BPM chitosan core (50,000–190,000 Da), were prepared. Conditions were set based on the correct Taylor cone formation, which was observed by a camera using IC Capture v2.4 software (The Imaging Source, Charlotte, NC, USA). The parameters for the correct formation of empty CS NPs and CS encapsulating rhIFNα-2b and rhIFN-γ were as follows: flow rate pump 1 (0.3 mL/h) pump 2 (0.2 mL/h), voltage difference injector (23.85 kV—collector 10.6 kV), and distance between the injector and collector 32.5 cm.

Nanoparticles are an organized system of agglomerates, and for the correct formation of solid (dry) NPs by electrospraying, a pH adjustment of the protein was required to achieve the correct self-assembly so that the system self-organizes, self-assembles, and self-agglomerates [75]. It is recommended that this pH modification is carried out by gradually increasing the pH from 0.5 and adjusting the conditions until the correct formation of the Taylor cone and the dry or solid NPs are visualized in the collector [76].

### 3.1. Physicochemical Characterization of Nanoformulations

The formulated nanoparticles were characterized according to physicochemical properties and structural attributes such as size, shape, composition, charge, surface chemistry, encapsulation efficiency, and release kinetics [77].

#### 3.1.1. Morphology and Size

**Figure 1:**
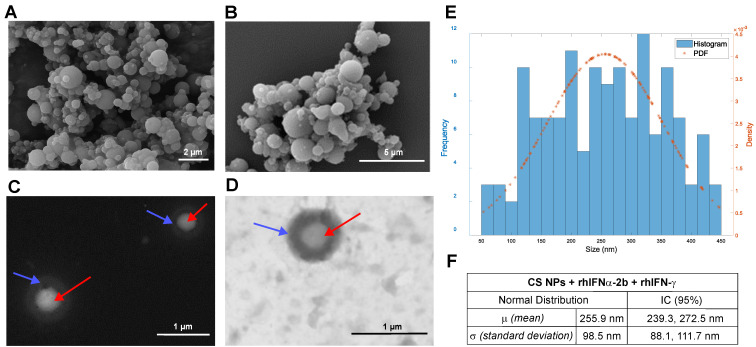
Characterization of empty and rhIFNα-2b + rhIFN-γ encapsulating CS NP morphology and size. (**A**) SEM micrograph of empty CS NPs. (**B**) SEM micrograph of CS NPs encapsulating rhIFNα-2b and rhIFN-γ. (**C**,**D**) STEM micrograph of the core-shell configuration of the nano-encapsulated CS formulation with the double structure (blue arrow: outer membrane; red arrow: inner membrane). (**E**) Histogram of the CS NPs with rhIFNα-2b and rhIFN-γ based on the determination of the probability density function. (**F**) Parameters of the normal distribution function.

The morphology was visualized by SEM and STEM micrographs. The NPs presented a spheroidal morphology of quasi-spherical sizes (Figure 1A,B). For CS NPs dispersed in ethyl acetate, two layers evidencing a “double membrane” (see arrows by STEM images) were identified (Figure 1C,D), demonstrating the core-shell structure of this formulation. The histograms showed that the different formulations were heterogeneous regarding size distribution. In addition, the assumption of whether or not the NPs’ measurement data followed a normal distribution was considered. The size values were taken with the filter at 450 nm, and a histogram with a Gaussian simulation (red-colored curve over the histogram) was made (Figure 1E,F and Figure S1). The Shapiro–Wilk test statistically corroborated that each formulation did not follow a normal distribution (Table 1).

Additionally, chitosan nanoparticles encapsulating rhIFNα-2b, which correspond to the core of the CS NPs formulation, were synthesized and characterized as a control for this formulation (Figure S2, Table S1).

Electron microscopy provided the most substantial evidence for determining the morphology of nanoparticles [78]. The size distribution, interfacial structure, compositional distribution, and phases of NPs that had a quasi-spherical spheroidal morphology of varying sizes were identified in the formulation [79]. The “double membrane” presence was determined, and a formulation combining the two interferons was obtained. Size measurements showed variability in the diameters of the resulting NPs, which are heterogeneous and do not follow a normal distribution [80]. Regulatory entities recommend combining multiple high-resolution orthogonal approaches to accurately evaluate size and distribution, similar to electron microscopy [81]. A second method corroborated previous results, DLS, which measured the diameter and stability of NPs in suspension and reported that the NPs were not monodisperse and tended toward polydispersity [82]. The dimensions of the nanoparticles designed in this study follow the accepted nanometrics for drugs containing nanomaterials, as outlined by the FDA [83]. The regulatory framework considers biological NPs to be attributable to nanoscale determinations up to one micrometer (1000 nm) in size.

#### 3.1.2. Mean Diameters and Zeta Potential

The DLS method evaluated the formulations and indicated that they are polydisperse with high distribution amplitude rather than monodisperse. The optical properties of the particles are unknown due to the large size dispersion observed by this method.

The measurement result by DLS should be considered indicative rather than definitive [84]. Solid formulations are out of range for this technique. The solution must be transparent or translucent due to using a visible light beam, which made the result difficult with this method [85]. The polymers, proteins, and formulations had different refractive indices, resulting in high polydispersity [86]. NPs with diameters greater than 200 nm possess excellent localization, facilitating migration into adjacent tissues [87]. The dimensions of the nanoparticles designed in this study are accepted as nanometrics conform to FDA [83], attributable to nanoscale determinations down to one micrometer in size (1000 nm).

All the methods used show that the NPs elaborated were heterogeneously polydisperse, and it could be statistically demonstrated that they do not follow a normal distribution. The methods of visualization of the results achieved with electron microscopy (histograms, FIJI©, MATLAB software) are very similar but present a wide standard deviation, indicating the polydispersity of the particles obtained. Despite microscopy methods, SEM and STEM are not comparable with DLS, which has a different working principle and yielded similar conclusions. With all these data, it is possible to suggest that, for this formulation, the most reliable results are those obtained by SEM or STEM, the only valid method to correctly calculate the size of nanoparticles. By electron microscopy, the empty CS NPs showed sizes averaging 209.3 ± 84.6 nm, and the CS NPs encapsulating the interferons averaged 255.9 ± 98.5 nm. For the DLS, the detected empty CS NPs’ size was 205.7–12.36 nm and 174.5–12.72 nm for those encapsulating interferons, with similar size determination by both methods (Table 1). The nanoparticles’ colloidal stability was analyzed by zeta potential as recommended for this analysis [88]. The zeta potential of the formulations should range between values above +25 mV or below −25 mV, which indicates the formulation’s colloidal stability [89], as is the case for the core-shell formulation obtained in this work (+24.5 ± 3.15 mV) [90]. The positive charge is explained by the presence of the amino groups on the chitosan molecules [91]. Formulations based on this cationic polymer have mucoadhesive properties, which are ideal for delivery to the intranasal mucosa [92]. Nanoparticles with this feature can enhance the transport of proteins across the epithelium and increase the residence time in the respiratory cavity due to electrostatic interactions with negatively charged sialic acid residues in the mucosa [93].

#### 3.1.3. Encapsulation Efficiency

The amount of protein loaded in a nanoparticulate system can be determined by calculating the encapsulation efficiency percentage of protein retained in the NPs relative to the total protein used for nanoencapsulation. In this assay, we analyzed the samples of the empty CS NPs and CS NPs encapsulating rhIFNα-2b and rhIFN-γ by SEC-HPLC chromatography to find the areas under the curve corresponding to each formulation element. The determination was performed, quantifying the free recombinant proteins (rhIFNα-2b and rhIFN-γ) in the supernatant of the nanoparticle batches using the TSK gel G2000SW matrix. The encapsulation efficiency of rhIFNα-2b and rhIFN-γ was calculated using the area values, resulting in a 76.7% encapsulation rate (Tables S2 and S3). This result seems adequate for excess protein in the reaction, increasing the amount of free protein that is not encapsulated and remains as free material in the reaction.

In the physicochemical characterization of nanoparticles, the encapsulation efficiency is considered a quality attribute estimating the drug loading capacity on the particles and the percentage of protein retained by the NPs relative to the total protein used for nanoencapsulation [94]. This parameter can be quantified through direct methods that evaluate the encapsulated drug and indirect methods that calculate the non-encapsulated drug [95]. The encapsulation efficiency analysis was performed using an indirect method, SEC-HPLC molecular exclusion high-performance liquid chromatography, which determined 76% EE. Similar results were obtained with this same method on porcine IFN-α for chitosan particles, with an EE of 74.96% [47]. The EE value and doses must be correlated with the drug release kinetics to achieve effective drug nanoformulation. Another indirect method, the commercial enzyme-linked immunosorbent assay (ELISA), has also been described. This method quantifies the amount of free drug in a chitosan formulation with rhIFNα-2b. The results of this study showed an EE of 99.9%, indicating a high-performance method [96]. It should be noted that the EE may not always accurately reflect the exact percentage of encapsulated drugs due to several factors that can affect its determination, such as polymers, structural stability after encapsulation, synthesis conditions of the NPs, and the concentration of the active ingredient [95].

#### 3.1.4. Characterization by Attenuated Total Reflectance Infrared Spectroscopy 

**Figure 2:**
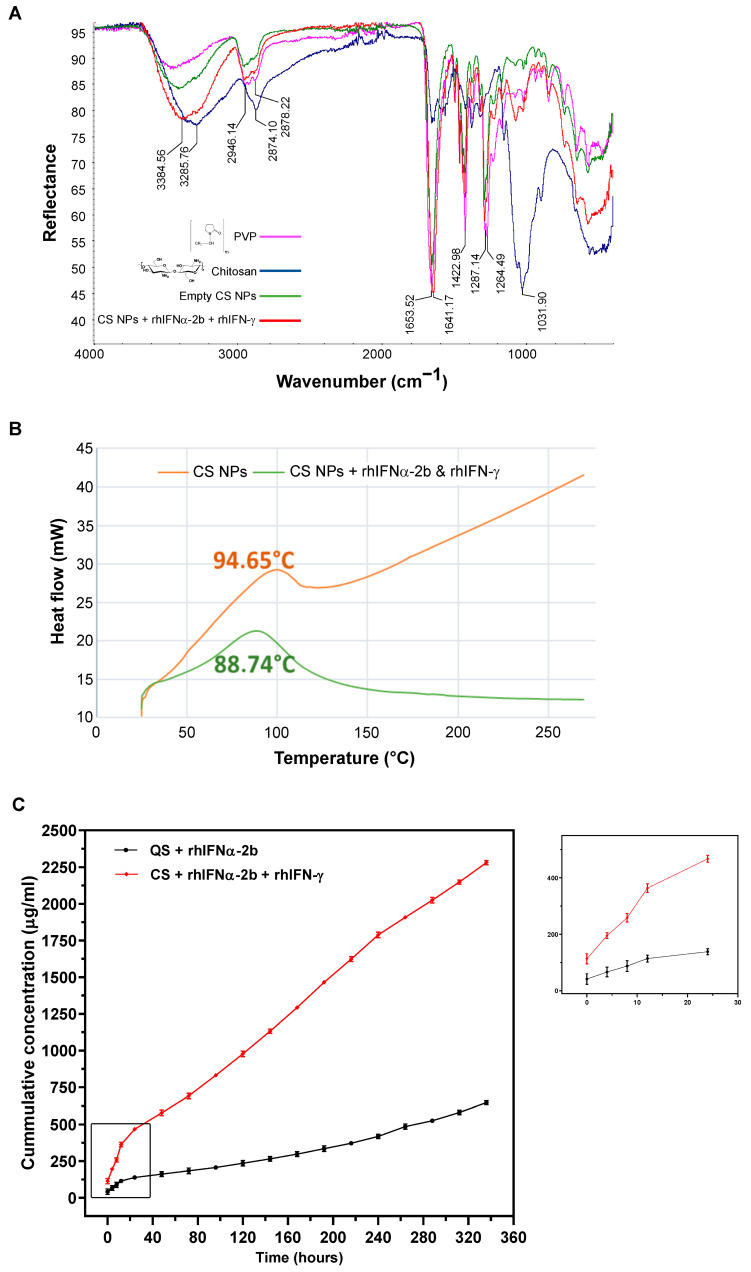
Physicochemical characterization of nanoformulations. (**A**) ATR-FTIR of empty CS and CS NPs encapsulating rhIFNa-2b and rhIFN-γ. (**B**) Thermal analysis of the CS NPs. The graph describes the temperatures reached in Differential Scanning Calorimetry for empty NPs and encapsulating rhIFNα-2b and rhIFN-γ. (**C**) Release kinetics of CS NPs + rhIFNα-2b and rhIFN-γ up to 14 days. Error bars represent mean ± SD (*n* = 3). The Micro BCA Protein Assay Kit quantification method was used.

We used attenuated total reflectance infrared spectroscopy to evaluate whether the encapsulated polymer shows infrared spectroscopy bands that differ from the base polymer, providing chemical information about the compounds present in the formulation [97]. The results showed a high level of similarity between the two formulations that both maintained the pure polymer characters, with slight shifts of some of the signals. There was no change in the chemical environment of the bonds after the encapsulation process, including the intermolecular interactions between the functional groups of the polymers (Figure 2A and Figure S3A). The analysis of the NPs by ATR-FTIR showed that the encapsulation process did not affect the polymers and that the peak signals of each pure polymer were maintained, with minor shifts typical of the encapsulation process without alterations in the polymeric structure. This study showed that nanoparticles maintained the typical fingerprints of pure polymers, with slight shifts in some signals due to the modification in the chemical environment of the bonds after the encapsulation process. Small changes in ATR-IR spectra suggest that the structure and function of the polymers were unaffected.

#### 3.1.5. Thermal Analysis by Differential Scanning Calorimetry (DSC)

Thermal stability was also investigated in polymers, empty nanoparticles, and protein encapsulation, a technique linked to stability, transition during encapsulation, and thermal degradation [98]. The glass transition temperature (Tg) was characterized to determine the stability and behavior of the polymers used and the formulations obtained at high temperatures. The empty NPs and the one encapsulating the interferons were exposed to temperatures from 25 °C to 270 °C at a heating rate of 20 °C/min. The thermogram of CS NPs with a 1% (*w*/*v*) LMW chitosan core coated with 10% (*w*/*v*) PVP-40000 showed a Tg of 94.65 °C (Figure 2B and Figure S3B). This result could be related to the plasticizing effect of the encapsulated proteins [99,100].

The thermal analysis results were as expected for amorphous and hygroscopic substances, with an endothermic effect of 90–140 °C due to the dehydration of the polymer [101,102]. The melting point of the endothermic peaks was 95.41 °C, indicating their crystalline nature. The Tg of the NPs encapsulating both interferons decreased. This analysis of the Tg of the biopolymers and their blends with each other predicts their dependence on water. It evidences the protective relationship between intra- and inter-macromolecular hydrogen bonds, dipole–dipole interactions, and plasticization functions [100]. The physical behavior of the proposed formulations and storage conditions were characterized [103]. Zhao, Duan [58] demonstrated slower and more stable thermal degradation during synthesis and encapsulation than polymer alone. The elaborated solid nanoparticles were stable at high temperatures with fluctuations very close to the Tg of the base polymers, allowing their use in other pharmaceutical forms, such as lyophilized or tablet forms, in the future.

#### 3.1.6. Evaluation of the In Vitro Nanoformulation Release Kinetics

One of the most critical challenges in evaluating the properties of nanoformulations is release kinetics [104]. It is crucial to consider the physiological environment accompanying this process in an in vivo model, which is the first pharmacokinetic approximation of the product to be evaluated [105]. The assessment of release kinetics allows for a more accurate identification of whether the drug is released slowly and sustained or wholly released [104]. The physiological conditions of the administration site may accelerate or decelerate the release of the encapsulated molecule. Physiological conditions of the nasal mucosa were estimated, with a pH of 6.8 corresponding to the target route of administration of these formulations, and a tissue temperature of 37 °C throughout the experiment [106]. In this experiment, the release of the CS formulation encapsulating rhIFNα-2b and rhIFN-γ was compared to the QS NPs encapsulating rhIFNα-2b, corresponding to the core of the CS formulation. The amount of protein included according to the NPs in the respective polymer solution (core 2 mg/mL of rhIFNα-2b and shell 1 mg/mL of each protein), the EE result, and the concentration of each protein were also considered. The results showed that both formulations after 14 days continue to release proteins, and this effect lasts over time (Figure 2C). The two formulations had a biphasic behavior, in which a sudden release is initially dominated by the diffusion phenomenon, where a high concentration of the proteins is released rapidly, followed by a slow and continuous release, with a greater effect for the CS NPs [107]. A double diffusion phenomenon was demonstrated for both formulations on the first day of the trial, with a large amount of protein released in a short period, which was released immediately after the nanoparticle was reconstituted, followed by a slow release in the following days. In the case of QS NPs, this behavior is due to the amount of unencapsulated free protein. However, for CS NPs, in addition to the free protein, the coat proteins are released immediately after the nanoparticle reconstitution. The mucoadhesive capacity of the chitosan represented the slow release [108]. The increased residence time causes the encapsulated drug to be released and absorbed slowly and gradually, traveling through the cells and effectively entering the circulation [109]. This sustained release is favorable for IFN-α because adverse effects are dose-dependent, and reaching active plasma concentrations at lower doses would ensure therapeutic efficacy, avoiding such effects [96]. Chitosan possesses a distinctive property that enables enhanced permeation through various mechanisms, including the transient opening of epithelial tight junctions to facilitate the penetration of hydrophilic molecules such as interferons [110]. The encapsulating matrix also influenced kinetics and PVP, contributing to the fast release due to its hydrophilic character and high solubility [38]. The outer coat polymer, polyvinylpyrrolidone, hydrolyses and rapidly releases interferons (IFN-α and IFN-γ).

Different trials have reported two-phase release kinetics: an abrupt release during the first few hours, followed by a slow and sustained release [111,112,113]. However, previous studies of encapsulation systems failed to show significant amounts of the encapsulated material during the first hours and only observed progressive and sustained release [39,47]. Some IFN-α formulations describe various methods of in vitro release kinetics, where the form of encapsulation and selection of the target tissue vary with relative success such as PLGA microspheres [114,115,116,117], copolymer micelles [118], and chitosan nanoparticles [96], with variability in the time of slow and sustained release or complete expulsion. With this experiment, the escape phenomenon of the encapsulated active ingredient could be predicted, and information on the in vitro–in vivo correlation could be provided [85].

### 3.2. Biological Characterization of the Nanoformulations

#### 3.2.1. Effect of Encapsulated Active Ingredients on Cell Viability

In addition to physicochemical characterization, the biological parameters of the formulations must be known, and toxicity must be established before in vivo testing. These toxic effects include cytokine production, inflammatory stimuli, and increased reactive oxygen and nitrogen species [119]. An MTT-based assay [120] showed the behavior of high-dose formulations in two cell lines: a HeLa cancer cell line and an HFF skin fibroblast line, establishing the effect of the formulation on cell viability. The HeLa cell line was used because of the activity of interferon-alpha in inhibiting its proliferation (apoptosis and antiproliferative activity of encapsulated interferons) [62].

**Figure 3:**
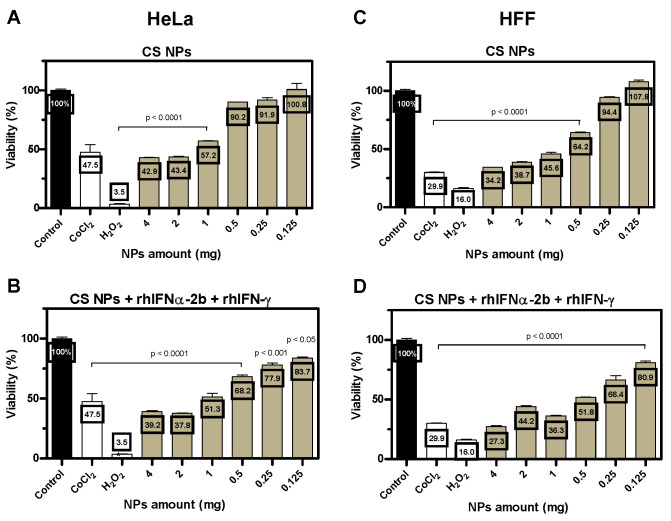

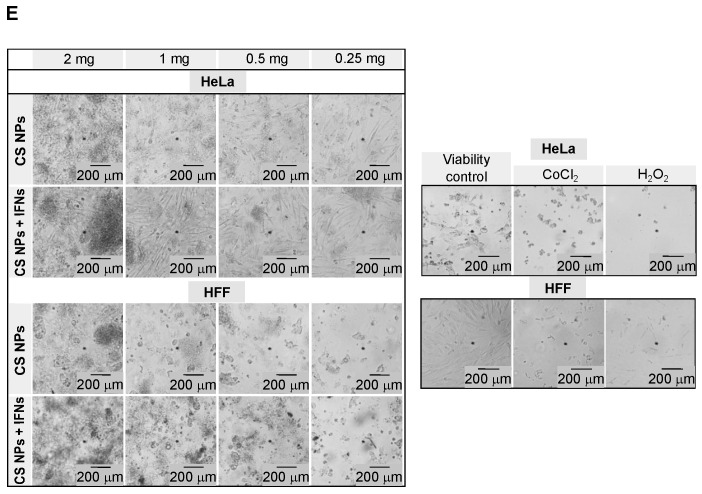
Viability of HeLa and HFF cell lines treated with CS NPs. (**A**) Viability of HeLa cells treated with empty CS NPs. (**B**) Viability of HeLa cells incubated with CS NPs + rhIFNα-2b + rhIFN-γ. (**C**) Viability of HFF cells treated with empty CS NPs. (**D**) Viability of HFF cells incubated with CS NPs + rhIFNα-2b + rhIFN-γ. (**E**) Micrographs of cells with the nanoformulations. Bars represent the mean ± SD (*n* = 3). Statistical significance was calculated using an independent *t*-test for comparison between empty and protein-containing NPs, and one-way repeated measures ANOVA with Bonferroni correction for the overall demonstration. The significance level set was 0.05.

A comparison was made between dilutions of empty versus protein-containing CS NPs at an initial concentration of 4 mg. The empty CS NP formulation in the HeLa line tended to decrease cell viability in those encapsulating interferons concerning the empty ones for both cell lines, but without statistical significance (Figure 3A,B). In the HFF line, there was statistical significance with an increase in cell viability of empty NPs for those encapsulating protein at dilutions of 0.5 mg (t = 28.35; *p* = 0.0224) and 0.125 mg (t = 14; *p* = 0.0454) (Figure 3C,D). For CS formulations encapsulating proteins, the decrease in cell viability was predominant for empty NPs with greater relevance in the HeLa cell line.

An evaluation of each formulation was also performed concerning the cell viability control.

Empty CS NPs: We found a decrease in cell viability compared to the control with significant differences for the HeLa line (F = 146.4; *p* < 0.0001) and the HFF line (F = 1353; *p* < 0.0001). Evaluating the dilutions compared to the control in HeLa with statistical significance for the 4 mg, 2 mg, and 1 mg dilutions (<60%). In HFF, the 4 mg, 2 mg, 1 mg, and 0.5 mg dilutions (<65%) had decreased viability relative to the control. The other dilutions had no significant differences (Figure 3A,C).

CS NPs encapsulating rhIFNα-2b and rhIFN-γ: Decrease in viability compared to the control with a significant difference: HeLa (F = 117.5; *p* < 0.0001) and HFF (F = 305.8; *p* < 0.0001). When evaluating the dilutions compared to the control in HeLa, there was a decrease in viability in 4 mg, 2 mg, and 1 mg (<60%) with statistical significance, and a tendency to increase cell viability was observed in the last three dilutions (>76%). HFF presented a decrease in viability in the 4 mg, 2 mg, and 0.5 dilutions (<55%), and in the last two dilutions, there is a tendency to increase (73%). The other dilutions had no significant differences (Figure 3B,D).

The formulation did not affect cell viability; there was a tendency for cell viability to increase as the dilutions increased. The antiproliferative effect of the encapsulated interferons in the HeLa cell line could be appreciated with a decrease in cell viability compared to the empty formulation.

This study used high starting doses, and the results showed that the formulations were non-toxic, as decreasing starting concentrations gradually increased cell viability. PVP increased cell proliferation at the latter concentrations, which had greater relevance to the formulation without active ingredients because PVP domains provide a suitable environment for cell growth [121,122]. Cell viability was lower in the protein-encapsulated formulations than in the empty formulations, especially in the HeLa cell line, showing the encapsulated interferons’ anticancer action [123]. This finding is related to the release kinetics assay that showed an initial abrupt release of the encapsulated proteins in the first hours and decreased cell viability on HeLa cells at early concentrations. The various forms of encapsulation reported in the literature for interferons have evaluated only the cell viability of the formulation [47,124] and not the effect of the biological activity of the active principle being encapsulated. In this work, we demonstrated both effects: the toxicity of the formulations in vitro and the antitumor activity of the IFNs in a cancer cell line.

#### 3.2.2. In Vitro Antiviral Biological Activity of Nanoformulations

In addition to the structural characteristics that define the properties of a nanoformulation, the determination of biological activity and structural stability are parameters to be considered in an encapsulated system [125]. The analysis of antiviral activity for the CS formulation encapsulating rhIFNα-2b and rhIFN-γ was performed independently for both interferons. The antiviral activity of the encapsulated proteins was then evaluated by comparing them with the respective standards (Figure S4). The cytotoxicity inhibition assay was used in HEp-2 cells exposed to the Mengo virus. The amount of protein added to each polymeric solution and the result of the EE were quantified.

**Figure 4:**
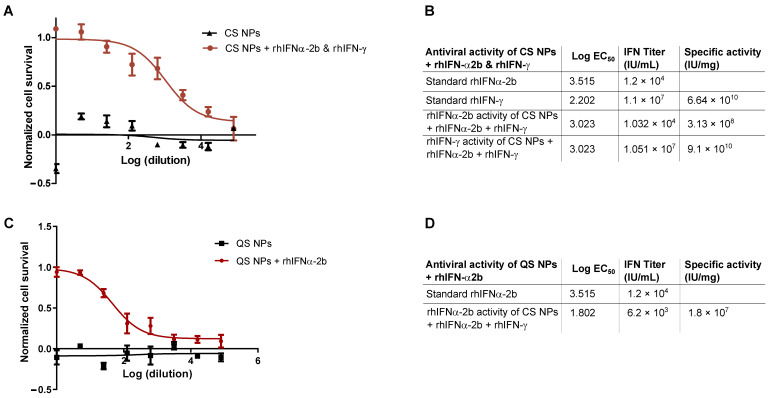
Evaluation of the antiviral activity of the nanoformulations. (**A**) Antiviral activity of interferons in the CS formulation. (**B**) Interferon titers and specific activity of the standards and the formulation. (**C**) Antiviral activity of rhIFNα-2b in the QS NPs. (**D**) IFN-α titers and specific activity of the standards and the formulation. Error bars represent mean ± SD (*n* = 3).

The titer of rhIFNα-2b equal to 1.032 × 10^4^ IU/mL was calculated for this formulation. Once the sample titer of rhIFNα-2b was obtained, the value was multiplied by 0.33 μg/mL, the concentration at which the initial samples of encapsulated rhIFNα-2b were evaluated. With this value, the specific activity of the rhIFNα-2b encapsulated in the CS NPs was determined to be equal to 3.13 × 10^8^ IU/mg (Figure 4A,B). For rhIFN-γ, the commercial rhIFN-γ standard was taken as a reference, with an initial concentration of 0.033 μg/mL, and the titer of rhIFN-γ in (IU)/mL was calculated with a value of 1.51 × 10^7^ IU/mL. The titer was multiplied by 0.033 μg/mL, the concentration at which the initial sample was evaluated before assay dilutions were performed (Figure 4B). The specific activity of the encapsulated rhIFN-γ was 9.1 × 10^10^ IU/mg (Figure 4A,B). The same procedure was employed for the determination of the antiviral activity of the control, which corresponds to the CS core (QS NPs encapsulating rhIFNα-2b) (see Figure 4C,D). By comparing these results with the titer and specific activity of the standard interferons as appropriate, it was evident that the two encapsulated proteins have similar antiviral activity to the standards.

The assay showed that the encapsulation process did not affect the specific activity of the interferons, and the encapsulated proteins maintained biological efficacy, with titer and particular activity in similar ranges to the standards. This method is very similar to other studies that refer to encapsulated interferons exerting antiviral action identical to the unencapsulated protein [96,126,127,128,129,130]. Investigations on IFN-α encapsulations revealed plasma concentrations comparable to the levels reached by free IFN via the systemic route [96,126]. In the same study, the molecule was detectable in plasma at 0.5 h with a concentration similar to that quantified after subcutaneous administration of free IFN-α [126]. These findings confirm that encapsulation protects IFNs and preserves activity in in vitro studies; however, the correlation between in vitro and in vivo studies should be explored [129].

#### 3.2.3. Confocal Microscopy Study of the Interaction of CS NPs

**Figure 5:**
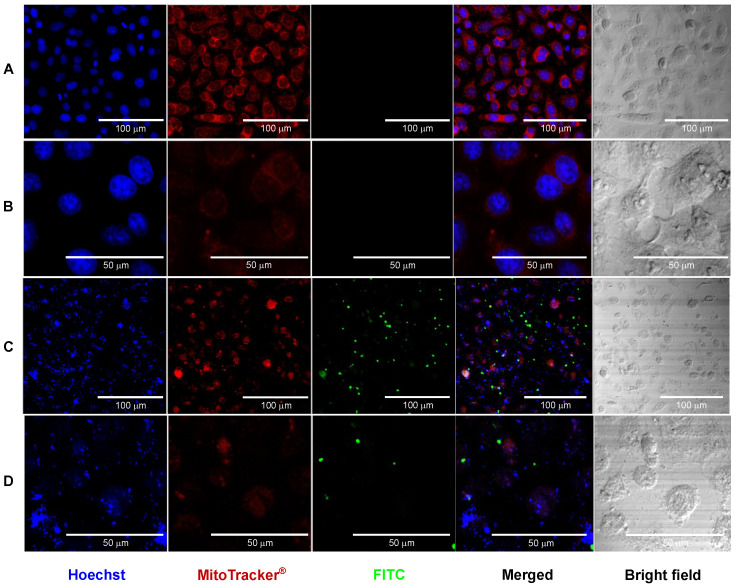
Confocal microscopy of the CS core-shell NPs formulation with FITC-labeled rhIFNα-2b. (**A**) Cells treated with empty CS NPs at 20× magnification. (**B**) Cells treated with empty CS NPs at 60× magnification. (**C**) CS NPs formulation encapsulating rhIFNα-2b and rhIFN-γ at 20× magnification. (**D**) CS NPs formulation encapsulating rhIFNα-2b and rhIFN-γ at 60× magnification.

Confocal microscopy stands out among the different experiments used to assess the dynamics of cellular uptake and localization of nanoparticles in vitro (i.e., cell membrane, cytoplasm, or nucleus) and relate it to biological activity [131]. The visualization technique uniquely combines minimally invasive optical access to the nanoscale internal structure and dynamics of cells and tissues with molecular detection specificity [132]. The behavior of CS NPs encapsulating interferon alpha and gamma interferon in interactions with live cells, specifically HEp-2 cells, was analyzed by confocal microscopy. Only the rhIFNα-2b protein was framed as it was represented in the shell and core of the NPs. At a concentration of 250 μg/mL, HEp-2 cells were treated for 24 h. After a confluence of 50 × 10^4^ empty CS NPs and CS NPs rhIFNα-2b + FITC (green fluorescence) and as a negative control, HEp-2 cells in DMEM medium (image not shown) were applied as treatments. At 24 h after treatment, nuclei were stained with Hoechst DNA intercalating dye (blue fluorescence) and mitochondria MitoTracker^®^ Red (red fluorescence). Images obtained on empty CS NPs show rounded nuclei with blue coloration corresponding to nucleic acids, material within the nuclear membrane, numerous mitochondria around the cytoplasm, and normal morphology with uniform co-localization. Well-defined cells and their organelles are distinguishable in co-localization and brightfield, and no FITC labeling exists (Figure 5A,B). We can affirm that the formulation is not toxic in the HEp-2 cell line. In the images of the CS NPs encapsulating the two interferons, Hoechst staining showed subcellular localization of nucleic acids and scattered nuclei showing non-rounded structures (blue deformed nucleic acid) pyknotic nuclei without nuclear membrane definition, with the display of chromatin condensation in the form of dark spots and a significant reduction in cytoplasmic volume. Mitochondria stained with MitoTracker^®^ Red are scattered in the cytoplasm; they are small and few have cytoplasmic disruption. The cells are in the process of apoptosis and cell death (Figure 5C,D). The FITC-labeled particles are outside the cells, and the protein does not enter the cytoplasm. In co-localization, nucleic acids are scattered without a membrane, cells are grainy, and apoptotic nuclei are small, fragmented, and highly textured, with decreased cell numbers compared to the control. Disruption and cell damage with potential anticancer effects are distinguished in brightfield cytoplasm.

The results showed that the empty formulation allowed a complete visualization of the labeled structures (nucleus and mitochondria), evidencing that the polymer combination was harmless to the cells. However, the formulation encapsulating the interferons reaffirmed that the protein was labeled with FITC and outside the cell, i.e., the nanoparticle was not internalized. To exert their action, interferons must be released into the extracellular medium because their receptors are located at the membrane level, and from there, the signaling cascade begins; in this design, to fulfill this action, the internalization of the NPs into the cells is not desired [133]. The most relevant aspect of this experiment was the contrast with the images of empty CS core-shell NPs and CS NPs encapsulating interferons. The latter formulation showed the development of an apoptotic process, with the destruction of the nucleus and nucleic acids and a decrease in mitochondria and programmed cell death, showing a potential anticancer effect. Type I IFNs influence NK cells’ maturation, homeostasis, and activation, eliminating tumor cells through other immune cells or the tumor microenvironment [134,135,136]. The antiproliferative effect of interferons has been widely described in in vitro and in vivo studies, including HEp-2 cells [137,138]. The therapeutic application of interferons is the most commonly used and approved for anticancer therapies [24]. This work demonstrates encapsulated interferons’ potential antiproliferative activity through cell viability and confocal microscopy. Therefore, this nanoformulation could have another therapeutic application.

We can conclude that the empty formulations combining these two polymers were innocuous for this cell line. The formulations encapsulated with both proteins again showed interferons’ antiproliferative cytotoxic effect on cancer cells.

#### 3.2.4. Stability of Nanoformulations Under Accelerated Conditions

**Figure 6:**
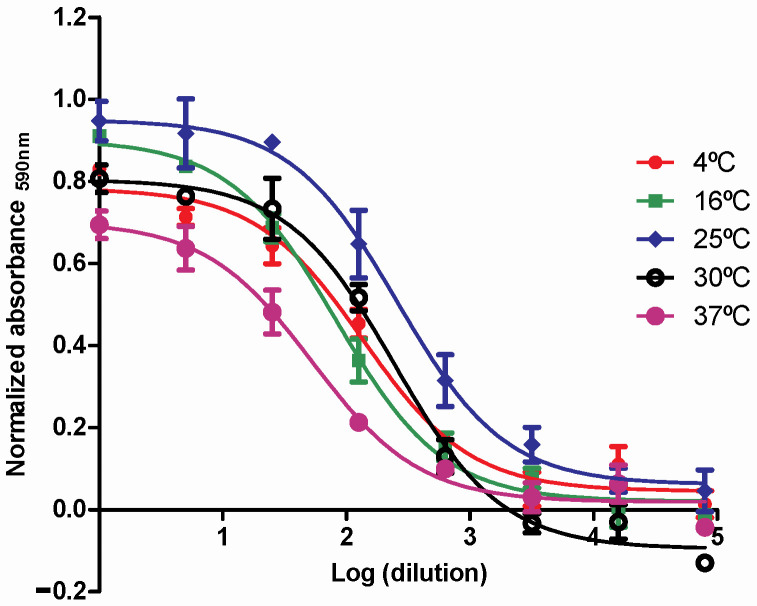
Stability under accelerated conditions of CS NPs + rhIFNα-2b + rhIFN-γ. Log (dilution) plot of the stability results of CS NPs encapsulating rhIFNα-2b and rhIFN-γ. Error bars represent mean ± SD (three replicates).

This experiment was performed to verify whether the nanoparticles maintained the biological activity of the encapsulated active principle at different Celsius degrees. Cells were treated with the CS NPs with rhIFNα-2b, rhIFN-γ, and the respective controls. A sigmoid curve was obtained that determined the value of the EC_50_ (Figure 6 and Figure S5). The analysis showed that the nanoparticles under stress conditions at different temperatures, 4 °C, 16 °C, 25 °C, 30 °C, and 37 °C remained stable for 18 days with excellent biological activity, which was found by calculating the EC_50_ (Tables S4–S6). The nanoparticles were stable under accelerated conditions for 18 days at all temperatures. There were no significant biological activity differences, so it can be inferred that encapsulation protects the active principle.

Stability is a critical aspect of ensuring the formulation’s safety and efficacy. It is closely related to the dosage [68] and should be evaluated under storage conditions [139]. In our work, we previously performed a thermal analysis using Differential Scanning Calorimetry of the formulations, which proved stable at temperatures close to 100 °C. Next, in the Stability of Nanoformulations Under Accelerated Conditions experiment, we evaluated the formulations’ stability by determining the encapsulated proteins’ antiviral activity and comparing them with the respective standards. The nanoparticles retained similar biological activity to the standards, demonstrated through the calculation of the EC_50_. The nanoparticles were stable under accelerated conditions for 18 days for all temperatures evaluated, so it can be inferred that the encapsulation protects the active principle from degradation with a positive effect on the pharmacokinetics [128].

### 3.3. Initial Safety Evaluation of NPs in Animal Models Such as Oryctolagus Cuniculus and Ovis aries

These nano-encapsulated formulations were designed for future human use; thus, initial toxicity had to be predicted in relation to the active ingredients. The experiment should be performed in an animal model that faithfully represents the pathophysiology of the human disorder and analyses the preliminary efficacy in vitro, followed by in vivo testing to determine the nanoparticle toxicity profile, especially if no previous data were available [140,141].

We evaluated the in vivo toxicity of the nanoformulation applied to the nasal route in two experiments: the first determined the mucosal irritant potential in rabbits and the second studied the safety on a sheep higher organism model.

In all in vitro experiments conducted in this research, the most successful formulation concerning the control, corresponding to the CS core, was the CS NPs, which encapsulate IFNs. Therefore, we selected these NPs for in vivo studies. It is important to note that the toxicological characterization of the active ingredients of these formulations has already been conducted, and their safety in animal models has been established. Both studies evaluated the treatment design, the effect on the entry route (harmlessness or irritability), and the first signs of nanoparticle distribution. For these trials, we used CS NPs encapsulating rhIFNα-2b and rhIFN-γ [140], and the intranasal human dose of IFN alpha 2b (Intron A) was considered at 1.7 × 10^8^ IU [142,143].

#### 3.3.1. Study of the Mucosal Irritant Potential of CS NPs in Rabbits

**Figure 7:**
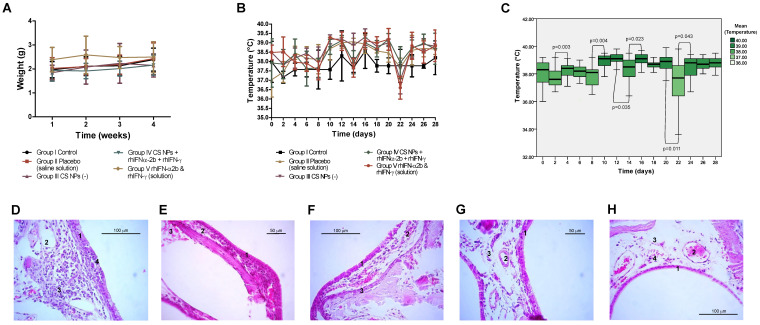
Mucosal irritability test in rabbits. (**A**) Relationship between organ weight and time for each treatment group. Data are expressed as mean ± SD (three replicates). Treatment groups were compared through a one-way ANOVA. The statistical significance set was α = 0.05. (**B**) Relationship between temperature °C and behavior according to the treatment groups. (**C**) The relationship between temperature in °C and behavior at intervals of every two days among the animals was analyzed using a non-parametric sign-test. The statistical significance set was α = 0.05. (**D**) Histopathological study, Group I: Control. Ciliated pseudostratified epithelium (1) with abundant chaliciform cells and a small erosion zone (4). Blood vessels with mild congestion (2), the lamina propria shows mild leukocyte infiltrate (3). (**E**) Histopathological study, Group II: Placebo. Ciliated pseudostratified epithelium (1) with normal appearance. Blood vessels with mild congestion (2) and a slight focal hemorrhage (3). (**F**) Histopathological study, Group III: empty CS NPs. Ciliated pseudostratified epithelium (1) with normal appearance. Blood vessels with mild congestion (2) and edema of lamina propria (3). (**G**) Histopathological study, Group IV: CS NPs + rh rhIFNα-2b + rhIFN-γ. Ciliated pseudostratified epithelium (1) with normal appearance. Blood vessels with mild congestion (2) and edema of lamina propria (3). (**H**) Histopathological study, Group V: rhIFNα-2b and rhIFN-γ in solution. Ciliated pseudostratified epithelium (1) with normal appearance. Blood vessels with mild congestion (2), mild edema of lamina propria (3), and leukocyte infiltrate (4).

The rabbits were evaluated for the weight variable for four weeks (Figure 7A), and the relationship between the body weight variable and the treatment groups per week was established. The control groups (Group I and Group II) had similar behavior in the four weeks. Group II Placebo (saline) as Group III (empty NPs) constituted controls for Group IV CS NPs (rhIFNα-2b-rhIFN-γ) and Group V (interferons in solution). Groups III and IV showed a trend toward weight recovery throughout the study. Group V started with higher weight values than the other groups and remained with similar figures until the end of the trial. The mean weight at week 1 vs week 4 was compared between the groups, and no significant differences were found (*p* > 0.0125) (Table S7). The proposed treatment scheme did not affect the weight of the animals, but rather, there was a trend toward weight gain without statistical significance. The average temperatures ranged between 37.3 °C and 38.9 °C during the 28 days of the trial (Figure 7B). The average temperature in each treatment group and at the different evaluation times did not differ significantly. Within each group, the oscillations of the initial and final temperatures were checked to determine if there were differences related to any group, with no significant differences. When comparing the temperatures at the different times measured in each of the groups, it was found that there was no group effect (*p* = 0.388), but there was a time effect (*p* = 0.000), and the temperature fluctuations were analyzed at intervals of every two days during the 28 days employing the Sign-Test (analysis aimed at evaluating the time effect). Significant differences in temperature fluctuations were found between the following intervals: days 2–4 (*p* = 0.003), 8–10 (*p* = 0.004), 14–16 (*p* = 0.023), and 22–24 (*p* = 0.043), with a significant increase in temperature. The variable was significantly decreased for the interval 12–14 (*p* = 0.035) and 20–22 (*p* = 0.011). The other intervals showed no significant differences (Figure 7C). Temperature variations were observed, which increased in the days of inoculation of the treatment. A response indicated the active principle’s activity and the treatment’s acceptability. Finally, basal temperature levels were recovered in the days following treatment. The animals had no behavioral changes or stigmata during the trial.

#### 3.3.2. Histopathological Study in Rabbits

The macroscopic evaluation analyzed the respiratory, digestive, urinary, circulatory, lymphatic, and skeletal muscle systems. Some internal organs were weighed for anatomic-morphological characterization (liver, kidney, heart, and lung). Organ weights were grouped according to treatment groups (Table S8). Liver (F = 0.35, *p* = 0.84), kidney (F = 0.29, *p* = 0.88), lung (F = 0.44, *p* = 0.77), and heart (F = 0.87, *p* = 0.51) weights showed no significant differences between treatment groups. No specific macroscopic lesions were observed in organs and systems.

Microscopic analysis was performed on the nasal mucosa, and criteria such as epithelium, leukocyte infiltration, vascular congestion, and edema were described as defining the state of the tissue. Most sections were regular, and the rest had minor damage to the epithelia, consisting of mild local erosions and cellular degeneration. All samples showed vascular congestion ranging from minimal to medium, typical of tissues with high blood supply. Unevenly distributed lesions between the treatment and control groups indicated no differences or pathological damage associated with the treatment. The observed focal lesions could be associated with environmental agents’ damage and not the treatments’ effects (Figure 7D–H).

We can conclude that the rabbits did not show irritability of the nasal mucosa, so the study was safe with no toxicity to the animals tested in the different groups. It was also demonstrated that the route of administration was safe for the formulation tested.

#### 3.3.3. Safety Study of CS NPs in Sheep

Of the 16 animals studied, 14 gained weight during the trial and 2 did not (sheep 1 group I and sheep 9 group IV interferons). When the body weight variable was evaluated statistically, it presented a normal distribution. The weight behavior during the study, when comparing the beginning and the end of the treatment (F = 0.858, *p* = 0.489; F = 0.703 *p* = 0.568), did not show significant differences in the two times evaluated (Table S9). For the average weights between the groups at each time evaluated, it was shown that there were no significant differences. Still, when comparing the average weight at the beginning vs. the end in each of the treatment groups, significant differences were found for Group 3 (CS NPs + rhIFNα-2b + rhIFN-γ) (*p* =0.037 < 0.05). However, the animals’ weight increased at the end of the treatment, which speaks in favor of the formulation’s safety (Table S9).

**Figure 8:**
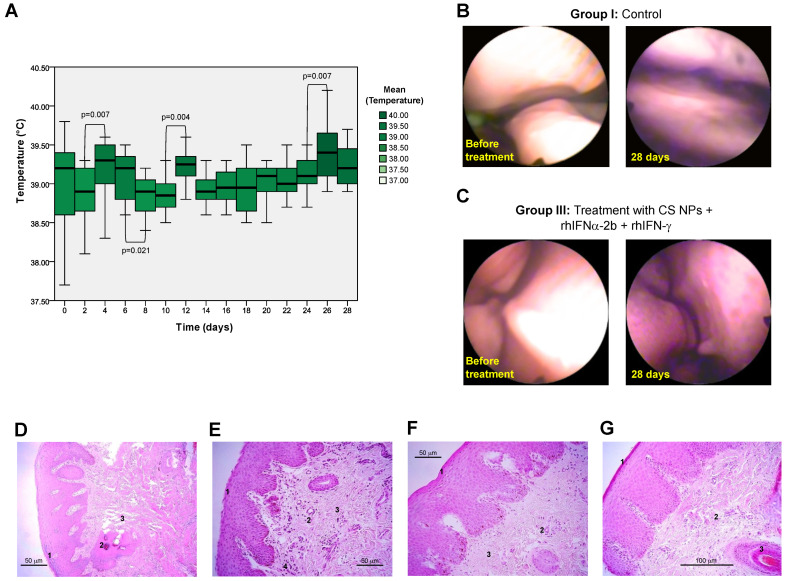
Safety study of the nanoformulations in higher organisms (sheep). (**A**) The relationship between temperature in °C and behavior at intervals of every two days among the animals was analyzed using a non-parametric sign-test. (**B**) Rhinoscopy of a sheep from Group I: Control. The nasal mucosa was observed without alterations, showing melanosis, a mucosal trait corresponding to the racial type, without other abnormalities. (**C**) Rhinoscopy of a sheep from Group III: Treatment with CS NPs + rhIFNα-2b + rhIFN-γ. It was observed that the nasal mucosa did not present alterations at any time. (**D**) Histopathological study, Group I: Control. Sample of nasal vestibule, slightly keratinized stratified epithelium (1). Mildly congestive blood vessels (2) and slight edema in the lamina propria (3). (**E**) Histopathological study, Group II: empty CS NPs. Sample of nasal vestibule, slightly keratinized stratified epithelium (1). Unaltered blood vessels (2) and normal-looking lamina propria (3). Mild leukocyte infiltrate (4). (**F**) Histopathological study, Group III: CS NPs + rhIFNα-2b + rhIFN-γ. Sample of nasal vestibule, slightly keratinized stratified epithelium (1). Unaltered blood vessels (2) and normal-looking lamina propria (3). (**G**) Histopathological study, Group IV: rhIFNα-2b and rhIFN-γ in solution. Sample of nasal vestibule, slightly keratinized stratified epithelium (1). Mild edema in lamina propria (2) and normal-looking hair follicle (3).

Concerning the average temperature between the groups at each of the times evaluated, it was found that there were no significant differences, there was no group effect, and there was no time effect. In the analysis of the Sign-Test to evaluate the time effect between intervals of every two days, significance was found in the following periods: 2–4 *(p* = 0.007), 10–12 (*p* = 0.004), and 24–26 (*p* = 0.007), with a significant increase in temperature (Figure 8A). For intervals 6 and 8 (*p* = 0.021), there was a significant decrease and a decreasing trend in the range 12–14. The other intervals showed no significant differences.

#### 3.3.4. Histopathological Study in Sheep

Macroscopic evaluation of the tissue was performed by nasal endoscopy (rhinoscopy) direct visualization of the nasal structures and nasosinusal anatomy. The sheep in the control group underwent rhinoscopy on days 0 and 28, and the rest of the groups underwent rhinoscopy on days 0, 14, and 28. No alterations were observed in any of the groups at the evaluated times. Several ewes presented melanosis, a mucosal trait corresponding to the racial type, without other abnormalities (Figure 8B,C).

Microscopic analysis showed no differences between the treatment and control groups, with no lesions or damage to the nasal mucosa attributed to the treatment (Figure 8D–G). The samples showed multiple artifacts caused by the sampling process and handling of the animals, consisting of compression necrosis, crushing, and deformation of the tissue and hemorrhages.

Selecting an appropriate toxicity model is a crucial step in predicting human biological responses [144]. However, there are few scientific articles on the type of animal species suitable for a nanoparticle that translates the intended effect to the biological activity evaluated [145,146,147].

In the first trial, the rabbit was selected for the mucosal irritability study because the nasal cavity presents superior characteristics to rodents, with similarity to the human nasal mucosa, such as the presence of hair follicles, transitional epithelium, and resistant squamous epithelium recommended to evaluate mucosal irritability [70,148]. The treatment regimens and results obtained in our trial were similar to those in previous studies [149,150,151], which used rabbits to evaluate intranasal powder formulations’ safety in repeated-dose toxicity studies. A toxicological study with polymeric nanoparticles used the rabbit and the nasal route in a pharmacokinetic assay [152]. However, no reports were found to determine mucosal irritability in this model, a novelty of our research, with suggestions for similar future studies.

Newer studies selected sheep as an animal model to evaluate the safety and efficacy of nanoformulations, which is considered a suitable model for the intranasal route [72,153,154]. The anatomical–functional characteristics of this system describe it as a human-like tissue, sufficiently spacious with an epithelial surface of 327 cm^2^ and a length of 18 cm, almost twice the human one (7.5 cm), that allows performing procedures and inoculating powder formulations [155].

The toxicity of nanoformulations is one of the most critical challenges limiting the clinical translation of NPs [147]. The two proposed in vivo studies evaluated the formulation’s safety equally with matching parameters, and similar results were obtained. The formulation proved to be non-toxic when assessing physiological conditions in both experiments. In both trials, temperature increased on the days of inoculation in the interferon groups but recovered on subsequent days without intervention. Fever is interferon therapy’s most commonly reported adverse event [156,157,158,159]. The toxicity of this formulation focused on local analysis associated with target organ damage, and a “hard variable” histopathological study of these experiments was performed. The analysis was conducted at the administration site using microscopic techniques in both studies. It showed no signs of inflammation, abnormal infiltration, or additional damage with the treatments applied compared to negative controls, and it was exposed that the therapy was harmless to the nasal mucosa. In vitro, studies such as cell viability assay and confocal microscopy allow the establishment of a correlation between in vitro and in vivo assays, a critical factor in the proposal of a new therapeutic option. In the safety evaluation of both experiments, we can summarize that the tested doses were not toxic in animal models. This statement supports the argument that this formulation remains a promising candidate for developing a successful antiviral. Our studies have only explored the safety of the CS core-coated NP formulation in two in vivo assays; therefore, the complete characterization of the preclinical profile is required to continue with the development of this product with studies such as acute toxicity, immunotoxicity, genotoxicity, carcinogenicity, and reproductive toxicity.

The selection of an appropriate administration route affects the proposed formulations. Based on current evidence, intranasal administration is the most attractive and novel route for encapsulated formulations of IFNs. This form of administration directly affects the three biological actions of these cytokines: antiviral, antiproliferative, and immunomodulatory [160]. From a pharmacological point of view, this route can be used for the non-invasive administration of drugs [161] due to the rapid absorption of most drugs, with high systemic concentrations, and the fact that the first-pass (hepatic) metabolism present in the oral route is avoided [162]. This has suggested the development of encapsulations for the interferons [163].

The use of biopharmaceuticals has contributed to shortening patients’ recovery time and improving their quality of life [28]. Recombinant proteins and antibodies are the most abundant therapeutic bioproducts on the market [164]. Among the recombinant therapeutic proteins, interferons have been widely supplied and demanded in the biopharmaceutical market [165], with 22 different formulations approved [24,166]. Currently, research is focused on obtaining new delivery systems for these biotherapeutics that provide adequate therapeutic concentrations, lower toxicity, and more excellent protection of the active principle [21,22]. Several formulations have been developed to encapsulate the IFNs [124,126,130,167], but for research purposes only in vitro and in vivo [30,128,168]. There is currently no formulation on the market that encapsulates interferons, so there is an opportunity to demonstrate that this system increases the therapeutic potential and safety of the drug [24].

Microparticles and nanoparticles are the most attractive formulations for intranasal interferon administration [169]. However, there is evidence in the case of interferons that microparticles affect the integrity of the active ingredient [170,171,172], showing low encapsulation efficiency [173,174,175] and abrupt or incomplete release of the protein [59,115,176] as well as reduced biological activity [114,177,178]. Nanoparticles are a promising encapsulation option for these proteins [175,179]. Nanometer-level encapsulations overcome the physical barrier of mucous membranes and penetrate effectively, protecting the active ingredient against biological and chemical degradation. Additionally, they offer higher stability, loading capacity, encapsulation efficiency, sustained release, and bioavailability [36]. Encapsulation enables improved pharmacological activity without increasing doses, with a more prolonged drug effect, higher bioavailability, and lower toxicity [180]. Nanoparticle systems are very successful as a tool for developing peptide and protein delivery, capable of improving the efficacy of established drugs and new molecules [181].

The proposed encapsulation system defines suitable therapeutic concentrations for type I and II interferons with a sustained and controllable release that preserves their structural and biological stability. The novelty of our work is given by the combined biological action of the interferons and the stepwise release system that allows a local and long-lasting release of IFN-α, potentiated with another cytokine that regulates the action, IFN-γ. Utilizing PVP and chitosan in a multilayer delivery system targeting the nasal mucosa represents a novel approach. The mechanical properties of the PVP, including flexibility and aqueous dispersion, are exhibited in the outer layer. Meanwhile, the core comprises chitosan, which exhibits desirable characteristics such as mucoadhesion and potential antimicrobial activity. This combination allows for achieving an optimal preventive and therapeutic response of immune system activation [29], which enhances the therapeutic index of macromolecules by creating a long-lasting delivery system [182]. This innovative approach aims to achieve the goals of controlled drug release, prolonged half-life, targeted delivery, and unsurpassed toxicity profile with nanoencapsulation procedures using biocompatible and biodegradable polymers [30].

## 4. Conclusions

This formulation is proposed as a novel drug delivery system and demonstrates its potential through physicochemical and biological characterization, with initial indications of safety as a pharmaceutical product. The encapsulation of rhIFNα-2b and rhIFN-γ in a core-shell structure by electrospray technique allowed for obtaining nanoparticles with controllable sizes, dispersions, and surface morphologies. The encapsulation efficiency was over 76.7%, prolonging the release of active compounds for more than two weeks in a simulated nasal mucosal environment. The encapsulation process did not affect the structure and function of the polymers used, and they were stable at high temperatures. Biological parameters such as the cytotoxicity of the formulation without affecting cell viability on two different cell lines and the anti-proliferative effect on the tumor line were determined. The encapsulation process did not affect the specific biological activity of the interferons, with ranges similar to standards. The cellular uptake and localization dynamics were also evaluated, confirming that the protein was not internalized and showed an apoptotic process with a potential anticancer effect. Stability was also investigated as a critical aspect to ensure the safety and efficacy of the formulation, showing that the nanoparticles were stable under accelerated conditions and that the encapsulation protected the drug from degradation. Safety studies in vivo models showed that the formulation was safe and caused no local or systemic damage in two animal models. Using the inhalation route as a drug delivery system in spray formulations was confirmed, avoiding first-pass metabolism.

We present a non-invasive intranasal inhaled dry powder formulation that does not require a cold chain and has the potential for self-administration, with efficacy and safety. These results are essential in an emerging or re-emerging pandemic of respiratory viral infections (e.g., SARS-CoV-2, influenza, or respiratory syncytial virus (RSV)). The effect of the particle is translated into a rapid and robust response against viral infections from the first day of application, which is crucial in situations where time is a critical factor for therapeutic success. The synergy of the two interferons enhances antiviral efficacy and may contribute to the inhibition of cell proliferation in mucosal oncology settings. This action has been demonstrated in two in vitro experiments, broadening this nanoformulation’s potential applications.

This innovative approach achieves controlled drug release, prolonged half-life, targeted delivery, and an optimal toxicity profile through nanoencapsulation by electrospraying and the use of biocompatible and biodegradable polymers. We have a novel drug with applications in multiple infectious, anticancer, and immunomodulatory diseases and we intend to continue its development due to its promising therapeutic potential.

## 5. Patents

The patent granted in Chile No. C-2023-51583 resulted from the work reported in this manuscript.

## Figures and Tables

**Table 1:** Size distribution of CS NPs by electron microscopy and Dynamic Light Scattering.

Nanoformulations	No. of Measured Parts	Average Particle Size	Histogram Filter 450 nm Fiji© Software	Shapiro–Wilk Statistical Test	DLS		
					Zeta Potential	Average Diameter	
Empty CS NPs	298	345.4 ± 352.2 nm	209.3 ± 84.6 nm	W = 0.953 *p* = 0.000	+25.9 ± 4.89 mV	205.7 nm 12.36 nm	92% (peak 1) 8% (peak 2)
CS NPs + rhIFNα-2b and rhIFN-γ	223	472.4 ± 337.6 nm	255.9 ± 98.5 nm	W = 0.977 *p* = 0.021	+24.5 ± 3.15 mV	174.5 nm 12.72 nm	75.4% (peak 1) 24.6% (peak 2)

Average of the measurements applying the 450 nm filter, Shapiro–Wilk statistical test, and the *p*-value for each formulation.

## Data Availability

The original contributions presented in the study are included in the article/supplementary material; further inquiries can be directed to the corresponding authors.

## Supplementary Materials

The following supporting information can be downloaded at https://www.mdpi.com/article/10.3390/pharmaceutics16111349/s1: Figure S1: Characterization of empty and rhIFNα-2b and rhIFN-γ encapsulating CS NPs morphology and size; Figure S2: Characterization of empty and rhIFNα-2b encapsulating QS NPs morphology and size; Table S1: Size distribution of QS NPs by electron microscopy and Dynamic Light Scattering (DLS); Table S2: Encapsulation efficiency results for CS NPs encapsulating rhIFNα-2b and rhIFN-γ; Table S3: Encapsulation efficiency results for QS NPs encapsulating rhIFNα-2b; Figure S3: Physicochemical characterization of QS nanoformulations; Figure S4: Evaluation of the antiviral activity of the nanoformulations; Table S4: Determination of antiviral activity under accelerated conditions for rhIFNα-2b from CS NPs encapsulating rhIFNα-2b and rhIFN-γ; Table S5: Determination of antiviral activity under accelerated conditions for rhIFN-γ from CS NPs encapsulating rhIFNα-2b and rhIFN-γ; Figure S5: Stability under accelerated conditions of QS NPs + rhIFNα-2b. Log (dilution) plot of the stability results of QS NPs encapsulating rhIFNα-2b; Table S6: Determination of antiviral activity under accelerated conditions for rhIFNα-2b from QS NPs encapsulating rhIFNα-2b; Table S7: Mucosal irritability test in rabbits. Animal weight behavior, Table S8: Mucosal irritability test in rabbits. Organ weight values for each treatment group; Table S9: Safety study of the nanoformulations in higher organisms (sheep). Animal weight behavior among the groups.
