# Supplementary material for: Multilayer Nanocarrier for the Codelivery of Interferons: A Promising Strategy for Biocompatible and Long-Acting Antiviral Treatment
Source: Pharmaceutics. 2024 Oct 22;16(11):1349. doi: 10.3390/pharmaceutics16111349 (PMC11597830; doi:10.3390/pharmaceutics16111349)
Supplement: Supplementary file 1 [file pharmaceutics-16-01349-s001.zip › pharmaceutics-3251554-supplementary.pdf]

Supplementary information

Multilayer Nanocarrier for the Codelivery of Interferons: A Promising Strategy for Biocompatible and Long-Acting Antiviral Treatment

Thelvia I. Ramos [1,2,*], Carlos A. Villacis-Aguirre [1], Felipe Sandoval Sandoval [1], Sarah Martin-Solano [2], Viana Manrique-Suárez [1], Hortensia Rodríguez [3], Leandro Santiago-Padilla [4], Alexis Debut [5], Carolina Gómez-Gaete [6], Marbel Torres Arias [2], Raquel Montesino [1], Emilio Lamazares [1], Ignacio Cabezas [7], Florence Hugues [7], Natalie C. Parra [1], Claudia Altamirano [8], Oliberto Sánchez Ramos [9], Nelson Santiago-Vispo [10] and Jorge R. Toledo [1,*]

1. Biotechnology and Biopharmaceutical Laboratory, Departamento de Fisiopatología, Facultad de Ciencias Biológicas, Universidad de Concepción, Víctor Lamas 1290, P.O. Box 160-C, Concepción 4030000, Chile; carlovillacis@udec.cl (C.A.V.-A.); felisandoval@udec.cl (F.S.S.); vmanrique@udec.cl (V.M.-S.); rmontesino@udec.cl (R.M.); elamazares@udec.cl (E.L.); natparra@udec.cl (N.C.P.)
2. Grupo de Investigación en Sanidad Animal y Humana (GISAH), Departamento de Ciencias de la Vida y la Agricultura, Universidad de las Fuerzas Armadas ESPE, Sangolquí 171103, Ecuador; ssmartin@espe.edu.ec (S.M.-S.); mmtorres@espe.edu.ec (M.T.A.)
3. Yachay Tech Medicinal Chemistry Research Group (MedChem-YT), School of Chemical Science and Engineering, Yachay University for Experimental Technology and Research (Yachay Tech), Yachay City of Knowledge, Urcuqui 100119, Ecuador; hmrodriguez@yachaytech.edu.ec
4. Max Delbrück Center for Molecular Medicine in the Helmholtz Association, 13125 Berlin, Germany; leandro.santiago@mdc-berlin.de
5. Laboratory of Characterization of Nanomaterials, Center of Nanoscience and Nanotecnology, Universidad de las Fuerzas Armadas ESPE, Sangolquí 171103, Ecuador; apdebut@espe.edu.ec
6. Department of Pharmacy, Faculty of Pharmacy, Universidad de Concepción, Concepción 4030000, Chile; cargomez@udec.cl
7. Clinical Sciences Department, Faculty of Veterinary Sciences, Universidad de Concepción, Vicente Méndez 595, Chillán 3780000, Chile; oscabeza@udec.cl (I.C.); flohugues@udec.cl (F.H.)
8. Escuela de Ingeniería Bioquímica, Facultad de Ingeniería, Pontificia Universidad Católica de Valparaíso, Av. Brasil 2085, Valparaíso 2362803, Chile; claudia.altamirano@pucv.cl
9. Laboratory of Recombinant Biopharmaceuticals, Departamento de Farmacología, Facultad de Ciencias Biológicas, Universidad de Concepción, Víctor Lamas 1290, P.O. Box 160-C, Concepción 4030000, Chile; osanchez@udec.cl
10. Bionatura Journal, Clinical Biotec SL, 28029 Madrid, Spain; santiago@clinicalbiotec.com

* Correspondence: tiramos@espe.edu.ec (T.I.R.); jotoledo@udec.cl (J.R.T.)

1. Results

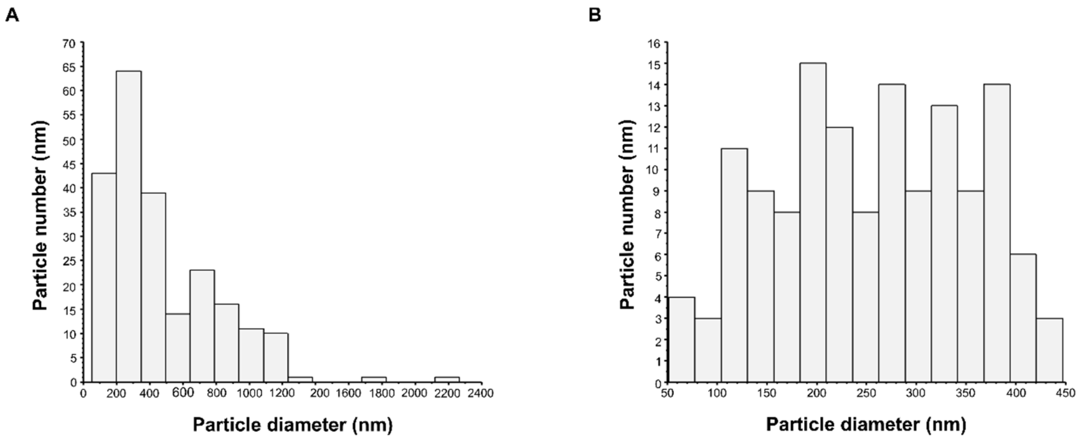

Figure S1. Characterization of empty and rhIFNα-2b and rhIFN-γ encapsulating CS NPs morphology and size. **A)** Histogram of the size distribution of the CS NPs with rhIFNα-2b and rhIFN-γ represented with the obtained data. **B)** Histogram of the size distribution of the CS NPs with rhIFNα-2b and rhIFN-γ by applying a filter at 450 nm to the data.

The histograms showed that the different formulations were heterogeneous regarding size distribution (Figure S1a). In addition to the above result, the assumption of whether or not the NPs measurement data followed a normal distribution was considered. The size values were taken with the filter at 450 nm (Figure S1b).

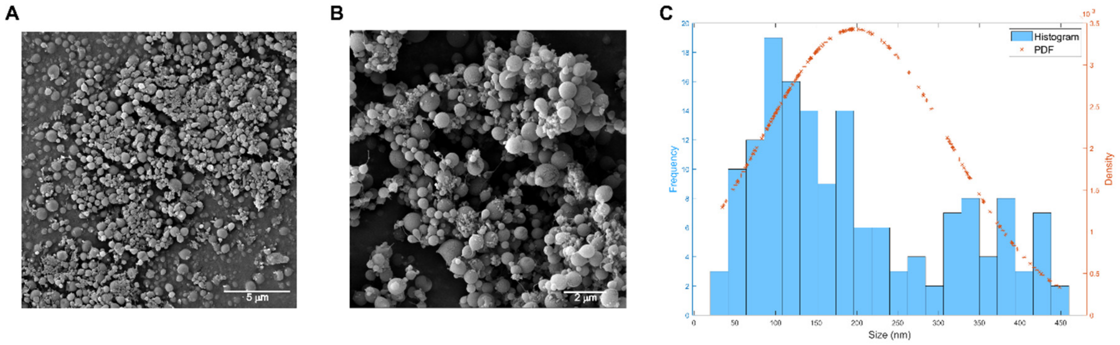

Figure S2. Characterization of empty and rhIFNα-2b encapsulating QS NPs morphology and size. **A)** SEM micrograph of empty QS NPs. **B)** SEM micrograph of QS NPs encapsulating rhIFNα-2b. **C)** Histogram of the CS NPs with rhIFNα-2b and rhIFN-γ based on the determination of the probability density function.

Table S1. Size distribution of QS NPs by electron microscopy and Dynamic Light Scattering (DLS).

**Nanoformulations**	**No. of measured parts**	**Average particle size**	**Histogram filter 450 nm *Fiji©* *Software***	***Shapiro Wilk* statistical test**	**DLS**		
					**Zeta potential**	**Average diameter**	
**Empty QS NPs**	234	301.2 ± 224.3 nm	188.2 ± 101.7 nm	W=0.911 p=0.000	+ 40.2 ± 3.73 mV	128.4 nm	100%
**QS NPs + rhIFNα-2b**	244	391.8 ± 340.5 nm	191.6 ± 91.1 nm	W=0.944 p=0.021	+ 40.7 ± 4.12 mV	207.9 nm	100%

Average of the measurements applying the 450 nm filter, Shapiro Wilk statistical test and the p value for each formulation.

Table S2. Encapsulation efficiency results for CS NPs encapsulating rhIFNα-2b and rhIFN-γ.

Total rhIFNs (rhIFNα-2b and rhIFN-γ)	326.3 Area [mV.Min]
Non-encapsulated rhIFNs	76 Area [mV.Min]
Encapsulated rhIFNs = total rhIFNs - non-encapsulated rhIFNs	250.3 Area [mV.Min]
EE = encapsulated rhIFNs / total rhIFNs	0.767085504
EE percentage	**76.7%**
Percentage of proteins at surface level	23.3%

Table S3. Encapsulation efficiency results for QS NPs encapsulating rhIFNα-2b.

Total rhIFNα-2b	296.5 Area [mV.Min]
Non-encapsulated rhIFNs	136 Area [mV.Min]
Encapsulated rhIFNs = total rhIFNs - non-encapsulated rhIFNs	160.5 Area [mV.Min]
EE = encapsulated rhIFNs / total rhIFNs	0.541315346
EE percentage	**54.1%**
Percentage of proteins at surface level	45.9%

*This encapsulation efficiency refers to the core of the CS NPs, composed of QS and rhIFNα-2b.

The amount of protein loaded in a nanoparticulate system can be determined by calculating the encapsulation efficiency percentage of protein retained in the NPs relative to the total protein used for nano encapsulation. The determination was performed using an indirect method, SEC-HPLC, quantifying the free recombinant proteins (rhIFNα-2b and rhIFN-γ) in the supernatant of the nanoparticle batches using *TSK gel G2000SW* matrix. The encapsulation efficiency analysis determined 76.7% for CS NPs encapsulating rhIFNα-2b and rhIFN-γ, and 54.1% for QS NPs encapsulating rhIFNα-2b.

A

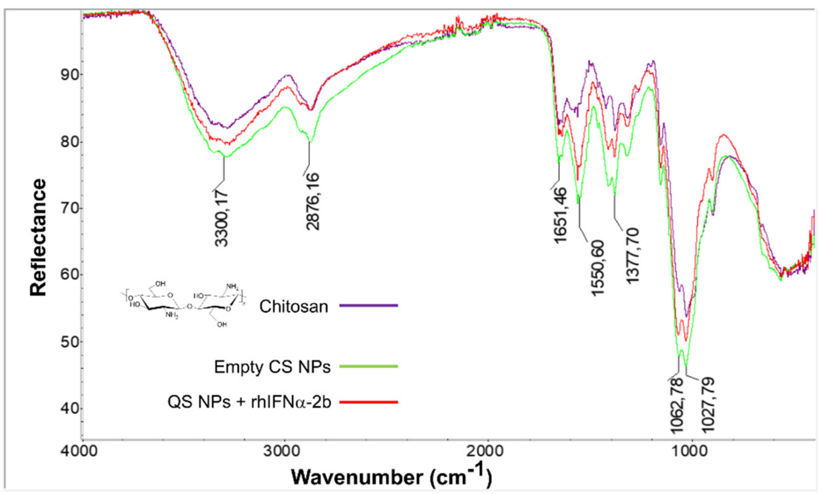

B

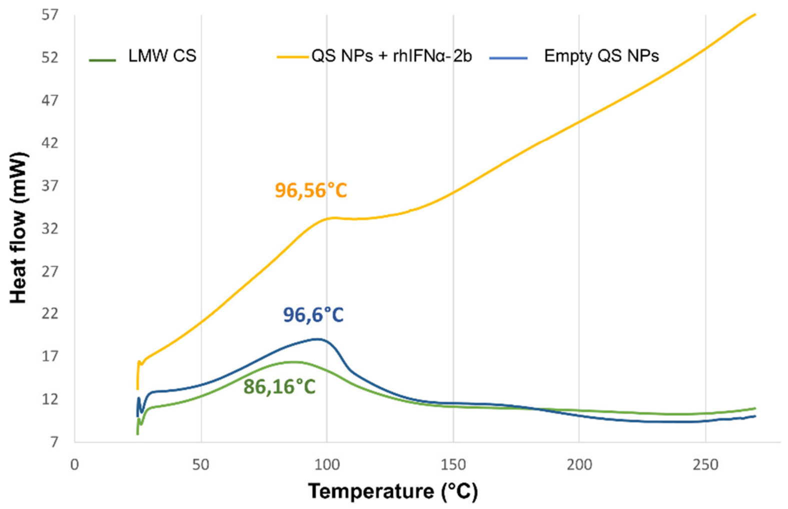

Figure S3. Physicochemical characterization of QS nanoformulations. **A)** ATR-FTIR of empty QS and QS NPs encapsulating rhIFNa-2b. **B)** Thermal analysis of the QS NPs. The graph describes the temperatures reached in Differential Scanning Calorimetry for empty NPs and encapsulating rhIFNα-2b.

For the formulation QS empty NPs and QS encapsulating rhIFNα-2b, corresponding to the core of the CS NPs, the ATR-FTIR spectra show that in the nanoparticles with chitosan the polymer signals have only small variations in displacement, even after the encapsulation process (Figure S3A). This implies that there is no structural variation in the formulation.

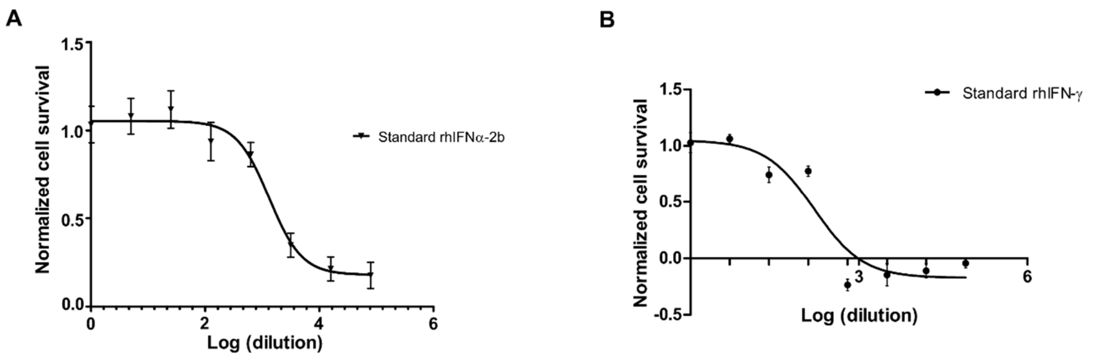

Figure S4. Evaluation of the antiviral activity of the nanoformulations. Log (Dilution) graph, to which HEp-2 cells were exposed to counteract the cytotoxic effect of Mengo virus vs. Cell survival, normalized with respect to 100% viability (cell control) and maximum lethality (virus control). **A)** Antiviral activity of standard rhIFNα-2b. **B)** Antiviral activity of standard rhIFN-γ.

Table S4. Determination of antiviral activity under accelerated conditions for rhIFNα-2b from CS NPs encapsulating rhIFNα-2b and rhIFN-γ.

**Antiviral activity of CS NPs + rhIFNα-2b & rhIFN-γ**	**Log EC_{50}**	**rhIFNα-2b titer (IU/mL)**	**rhIFNα-2b specific activity (IU/mg)**
Standard rhIFNα-2b	3.515	1.2×10^4	
rhIFNα-2b activity of CS NPs + rhIFNα-2b & rhIFN-γ. 4°C	2.133	7.2×10^3	2.2×10^8
rhIFNα-2b activity of CS NPs + rhIFNα-2b & rhIFN-γ.16°C	1.918	6.5×10^3	2.0×10^8
rhIFNα-2b activity of CS NPs + rhIFNα-2b & rhIFN-γ. 25°C	2.413	8.2×10^3	2.5×10^8
rhIFNα-2b activity of CS NPs + rhIFNα-2b & rhIFN-γ. 30°C	2.388	8.1×10^3	2.5×10^8
rhIFNα-2b activity of CS NPs + rhIFNα-2b & rhIFN-γ. 37°C	1.724	5.9×10^3	1.7×10^8

Table S5. Determination of antiviral activity under accelerated conditions for rhIFN-γ from CS NPs encapsulating rhIFNα-2b and rhIFN-γ.

**Antiviral activity of CS NPs + rhIFNα-2b & rhIFN-γ**	**Log EC_{50}**	**rhIFNα-2b titer (IU/mL)**	**rhIFN-γ Specific activity (IU/mg)**
Standard rhIFN-γ	2.202	1.1×10^7	6.6×10^{10}
rhIFN-γ activity of CS NPs + rhIFNα-2b & rhIFN-γ. 4°C	2.133	1.0×10^7	6.4×10^{10}
rhIFN-γ activity of CS NPs + rhIFNα-2b & rhIFN-γ. 16°C	1.918	9.5×10^6	5.7×10^{10}
rhIFN-γ activity of CS NPs + rhIFNα-2b & rhIFN-γ. 25°C	2.413	1.2×10^7	7.3×10^{10}
rhIFN-γ activity of CS NPs + rhIFNα-2b & rhIFN-γ. 30°C	2.388	1.2×10^7	7.2×10^{10}
rhIFN-γ activity of CS NPs + rhIFNα-2b & rhIFN-γ. 37°C	1.724	8.0×10^6	5.2×10^{10}

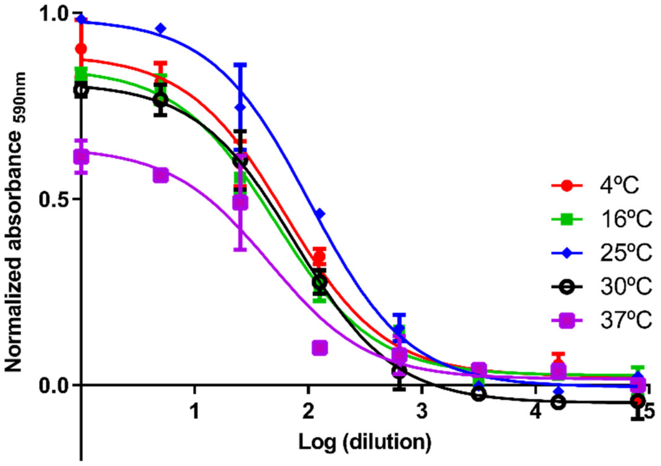

Figure S5. Stability under accelerated conditions of QS NPs + rhIFNα-2b. Log (dilution) plot of the stability results of QS NPs encapsulating rhIFNα-2b. Error bars represent mean ± SD (three replicates).

Table S6. Determination of antiviral activity under accelerated conditions for rhIFNα-2b from QS NPs encapsulating rhIFNα-2b.

Antiviral activity of QS NPs + rhIFNα-2b	Log EC50	rhIFNα-2b titre (IU)/mL	rhIFNα-2b specific activity (IU)/mg
Standard rhIFNα-2b	3.515	1.2×10^4	
rhIFNα-2b activity of QS NPs + rhIFNα-2b. 4°C	1.802	6.2×10^3	1.8×10^8
rhIFNα-2b activity of QS NPs + rhIFNα-2b. 16°C	1.710	5.8×10^3	1.8×10^8
rhIFNα-2b activity of QS NPs + rhIFNα-2b. 25°C	2.015	6.9×10^3	2×10^8
rhIFNα-2b activity of QS NPs + rhIFNα-2b. 30°C	1.885	6.4×10^3	1.9×10^8
rhIFNα-2b activity of QS NPs + rhIFNα-2b. 37°C	1.663	5.7×10^3	1.7×10^8

Table S7. Mucosal irritability test in rabbits. Animal weight behaviour: Data are expressed as mean ± SD. Treatment groups were compared through repeated measures ANOVA, as there were more than two evaluation times, and Bonferroni error correction was considered. In addition, a student's t-test for dependent samples was performed. The statistical significance set was $\alpha = 0.05$.

**Weight (g) / Treatment**		**Group I**	**Group II**	**Group III**	**Group IV**	**Group V**	**ANOVA F (p)**
**Total**		**5**	**5**	**5**	**5**	**5**	
Week 1 / Mean ± SD		1.97 ± 0.34	2.02 ± 0.45	1.84±0.31	1.94 ± 0.45	2.39 ± 0.51	1.234 (0.328)
Week 2 / Mean ± SD		2.11 ± 0.39	2.10 ± 0.34	2.08 ± 0.71	0.91 ± 0.32	2.59 ± 0.78	1.079 (0.393)
Week 3 / Mean ± SD		2.13 ± 0.40	2.18 ± 0.40	2.23 ± 0.83	2.01 ± 0.31	2.48 ± 0.46	0.556 (0.697)
Week 4 / Mean ± SD		2.39 ± 0.48	2.43 ± 0.66	2.14 ± 0.48	2.16 ± 0.42	2.50 ± 0.64	0.434 (0.782)
**p *(t-test)***	Week 1 *vs* Week 4	0.022	0.067	0.053	0.307	0.516	

The rabbits were evaluated for the weight variable for 4 weeks, and the relationship between the body weight variable and the treatment groups per week was established. The control groups (Group I and Group II) had similar behaviour in the four weeks. Group II Placebo (saline) as Group III (empty NPs) constituted controls for Group IV CS NPs (rhIFNα-2b-rhIFN-γ) and Group V (interferons in solution). Groups III and IV showed a trend toward weight recovery throughout the study. Group V started with higher weight values than the other groups and remained with similar figures until the end of the trial. The mean weight at week 1 vs week 4 was compared between the groups, and no significant differences were found ($p > 0.0125$) (Data not shown). The proposed treatment scheme did not affect the weight of the animals, but rather, there was a trend towards weight gain without statistical significance.

Table S8. Mucosal irritability test in rabbits. Organ weight values for each treatment group. Data are expressed as mean ± SD.

Organs/ groups	Group I Control (g)	Group II Placebo (saline solution) (g)	Group III Empty CS NPs (g)	Group IV CS NPs + rhIFNα-2b & rhIFN-γ (g)	Group V rhIFNα-2b & rhIFN-γ (g)
**n**	**5**	**5**	**5**	**5**	**5**
Liver	69.2 ± 10.03	71.8 ± 13.24	67.0 ± 13.21	68.0 ± 13.40	75.2 ± 13.16
Kidneys	12.2 ± 1.79	12.2 ± 2.28	11.6 ± 2.51	11.8 ± 2.49	13.0 ± 2.35
Lung	5.0 ± 0.71	4.8 ± 0.84	4.4 ± 0.89	4.6 ± 0.89	5.0 ± 1.0
Heart	5.0 ± 0.71	5.0 ± 0.71	4.6 ± 0.89	5.0 ± 1.22	5.6 ± 0.89

This test analysed the respiratory, digestive, urinary, circulatory, lymphatic, and skeletal muscle systems. Some internal organs were weighed for anatomic-morphological characterization (liver, kidney, heart, and lung). Organ weights were grouped according to treatment groups. Liver ($F=0.35$, $p=0.84$); kidney ($F=0.29$, $p=0.88$), lung ($F= 0.44$ $p=0.77$) and heart ($F=0.87$, $p=0.51$) weights showed no significant differences between treatment groups. No specific macroscopic lesions were observed in organs and systems.

Table S9. Safety study of the nanoformulations in higher organisms (sheep). Animal weight behaviour among the groups. Data are expressed as mean ± SD. Treatment groups were compared through repeated measures ANOVA with Bonferroni correction. In addition, a dependent t-test was performed. The statistical significance set was α = 0.05.

**Weight (g) / Treatment**		**Group I**	**Group II**	**Group III**	**Group IV**	**ANOVA F (p)**
**Total**		**4**	**4**	**4**	**4**	
Start		52.2 ± 8.7	45.8 ± 8.3	52.2 ± 5.9	48.0 ± 3.9	0.858 (0.489)
End		52.6 ± 9.7	46.9 ± 8.7	53.4 ± 5.5	48.7 ± 4.4	0.703 (0.568)
**p *(t-test)***	Start *vs* End	0.657	0.047	**0.037**	0.102	

The weight behaviour during the study when comparing the beginning and the end of the treatment (F=0.858, p= 0.489; F=0.703 p= 0.568) did not show significant differences in the two times evaluated. For the average weights between the groups at each time evaluated, it was shown that there were no significant differences. Still, when comparing the average weight at the beginning vs. the end in each of the treatment groups, significant differences were found for Group 3 (CS NPs + rhIFNα-2b + rhIFN-γ) ($p=0.037 < 0.05$). However, the animals weight increased at the end of the treatment, which speaks in favour of the formulation's safety.
